# Crystal Structure of 4-Hydroxyphenylpyruvate Dioxygenase in Complex with Substrate Reveals a New Starting Point for Herbicide Discovery

**DOI:** 10.34133/2019/2602414

**Published:** 2019-07-08

**Authors:** Hong-Yan Lin, Xi Chen, Jia-Nan Chen, Da-Wei Wang, Feng-Xu Wu, Song-Yun Lin, Chang-Guo Zhan, Jia-Wei Wu, Wen-Chao Yang, Guang-Fu Yang

**Affiliations:** ^1^Key Laboratory of Pesticide & Chemical Biology of Ministry of Education, International Joint Research Center for Intelligent Biosensor Technology and Health, College of Chemistry, Chemical Biology Center, Central China Normal University, Wuhan 430079, China; ^2^MOE Key Laboratory of Protein Sciences, Tsinghua-Peking Center for Life Sciences, School of Life Sciences, Tsinghua University, Beijing 100084, China; ^3^College of Chemistry and Material Science, South-Central University for Nationalities, Wuhan 430074, China; ^4^Department of Pharmaceutical Sciences, College of Pharmacy, University of Kentucky, 789 South Limestone Street, Lexington, KY 40536, USA; ^5^Collaborative Innovation Center of Chemical Science and Engineering, Tianjin 30071, China

## Abstract

4-Hydroxyphenylpyruvate dioxygenase (HPPD) is a promising target for drug and pesticide discovery. The unknown binding mode of substrate is still a big challenge for the understanding of enzymatic reaction mechanism and novel HPPD inhibitor design. Herein, we determined the first crystal structure of* Arabidopsis thaliana* HPPD (*At*HPPD) in complex with its natural substrate (HPPA) at a resolution of 2.80 Å. Then, combination of hybrid quantum mechanics/molecular mechanics (QM/MM) calculations confirmed that HPPA takes keto rather than enol form inside the HPPD active pocket. Subsequent site-directed mutagenesis and kinetic analysis further showed that residues (Phe424, Asn423, Glu394, Gln307, Asn282, and Ser267) played important roles in substrate binding and catalytic cycle. Structural comparison between HPPA-*At*HPPD and holo-*At*HPPD revealed that Gln293 underwent a remarkable rotation upon the HPPA binding and formed H-bond network of Ser267-Asn282-Gln307-Gln293, resulting in the transformation of HPPD from an inactive state to active state. Finally, taking the conformation change of Gln293 as a target, we proposed a new strategy of blocking the transformation of HPPD from inactive state to active state to design a novel inhibitor with *K*_i_ value of 24.10 nM towards* At*HPPD. The inhibitor has entered into industry development as the first selective herbicide used for the weed control in sorghum field. The crystal structure of* At*HPPD in complex with the inhibitor (2.40 Å) confirmed the rationality of the design strategy. We believe that the present work provides a new starting point for the understanding of enzymatic reaction mechanism and the design of next generation HPPD inhibitors.

## 1. Introduction

*α*-Keto acid dependent dioxygenases constitute the largest family of mononuclear nonheme iron enzymes that activate dioxygen to catalyze a wide variety of key biochemical transformations with pharmaceutical, agrochemical, and environmental significance [1–4]. As an important member, 4-hydroxyphenylpyruvate dioxygenase (HPPD) catalyzes the complex transformation of 4-hydroxyphenylpyruvate acid (HPPA) to homogentisic acid (HGA), a key step in the tyrosine catabolic pathway that is common to all the aerobic organisms. In plants, HGA is a key intermediate in the biosynthesis of tocopherols and plastoquinones, which are essential compounds for the photosynthesis [5–7]. Inhibition of the enzyme results in foliage bleaching of plants and eventually death [8–11], and thus HPPD inhibitors have been developed as herbicides for many years [12–16]. In mammals, HPPD plays a central role in the catabolism of tyrosine, and variants have been directly or indirectly involved in a number of metabolic disorders [17, 18]. A well-known HPPD inhibitor, 2-(2-nitro-4-trifluoromethyl-benzoyl)-1,3-cyclohexanedione (NTBC), has been approved by FDA for the treatment of type I tyrosinemia and alkaptonuria [19–22].

It has been known that the HPPD inhibitors for the treatment of human inherited diseases bind HPPD by competing with the substrate HPPA. Although significant advancement on the structure and catalytic mechanism of HPPD have been achieved [23–29], the exact binding model of its natural substrate HPPA remains unclear, which is critical to understand the interaction mechanism of inhibitors and molecular design of novel inhibitors. There are several crystal structures of HPPD in complex with (PDB codes: 1T47, 1SQI, 1TFZ, 1TG5, 5DHW, 5CTO, and 5YWG) or without (PDB codes: 1CJX, 1SP8, 1SP9, 1SQD, 3ISQ, and 5EC3) inhibitors [30–34]. In our recent study, we studied the mechanistic insights on the slow binding mechanism of HPPD inhibitors by combining the experimental and computational techniques [34]. Since all the reported HPPD inhibitors are competitive inhibitors, the exact binding mode of the substrate should be very helpful for the structure-based inhibitor design. According to the indirect information provided by the available structural data, three plausible HPPD-substrate interaction models ([Supplementary-material supplementary-material-1]) were proposed [25, 31, 32, 35], among which one was inspired by the first crystal structure of* pseudomonas fluorescens* HPPD (PDB code: 1CJX), two other models were hypothesized according to the crystal structure of* Streptomyces avermitilis* HPPD (*Sav*HPPD, PDB code: 1T47) and the p-hydroxymandelate (HMA) binding mode in hydroxymandelate synthase (HMS, ~40% sequence similarity with HPPD and share the same substrate HPPA). Apparently, these three binding modes are informative, but they did not reflect the real binding conformation of HPPA in active site of HPPD. In addition, because HPPA has two tautomeric forms, another key issue is whether the enzyme is bound by enol or ketone form. It was suggested that the keto tautomer is the active form of the substrate, while the enol tautomer binds to enzyme-bound catalytic metal ion and inhibits the reaction [36, 37]. Thus, the tautomeric forms of HPPA bound in the active site of HPPD have important implications for substrate turnover.

As a continuation of our work on the fundamental chemical biology study on the HPPD, herein we reported the first crystal structure of HPPA-bound* At*HPPD and determined the real existing isoform of HPPA in the active site by combining hybrid quantum mechanics/molecular mechanics (QM/MM) calculations. Subsequently, we identified the key residues involved in HPPD catalysis by combining the enzymatic kinetics and site-directed mutation. Finally, we carried out a structure-based design and successfully discovered a novel inhibitor (experimental code is Y13161) as the first selective herbicide for the weed control in sorghum field. This work provides a representative example of structure-and-mechanism-based design of pesticide.

## 2. Results

### 2.1. Crystal Structure of AtHPPD in Complex with HPPA

**Figure 1:**
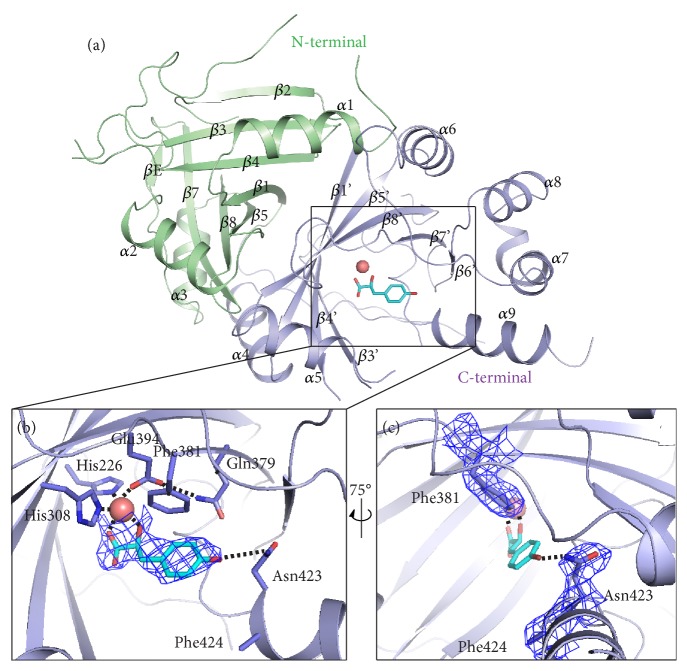
Crystal structure analysis of* At*HPPD-HPPA complex. (a) Overall structure of* At*HPPD-HPPA complex. The* C-* and* N*-terminal *β*-barrel domains are colored in light blue and green, respectively. The metal atom is shown as a deep salmon sphere, and HPPA was displayed in cyan. (b) An expanded view of the* At*HPPD active site showing an H-bond interaction between the HPPA and the residues nearby. (c) The expanded view of the* At*HPPD active site related to B with a 75° rotation around a vertical axis. Light blue electron densities (2Fo–Fc map) correspond to HPPA and key residues contoured at 1.0 *σ*. The key residues are shown in sticks, while the chelation with the metal ion and the H-bonds are indicated with black dash lines.

Wild type and mutants of recombinant* At*HPPD were overexpressed in* E. coli* BL21 (DE3) and sequentially purified by Ni-NTA affinity chromatography, anion exchange chromatography, and size exclusion chromatography. The purified* At*HPPD was analyzed by the size exclusion chromatography. The 11.5 to 17.5 mL fraction, which exhibited the highest purity protein (determined by the SDS-PAGE), was used in crystallization studies ([Supplementary-material supplementary-material-1]). Similar to the other members of the dioxygenase family, the catalytic center contains Fe^2+^ as an essential metal for HPPD activity. The purified HPPD displayed a very low activity that was restored upon addition of exogenous Fe^2+^ to the assay buffer, but 630 *μ*M Co^2+^ rather than Fe^2+^ was added during crystallization. However, since the identity of the metal is not rigorously established, it was referred to the active site metal in all the four chains (A, B, C, and D) as M in the previous report [30]. Here, crystals were obtained by the hanging drop vapor diffusion method. After screening for optimal growth conditions, crystals of purified HPPD (315 *μ*M) were grown in the presence of 650 *μ*M cobalt (II) chloride and 900 *μ*M HPPA. The crystal structure of the* At*HPPD-HPPA complex (PDB ID: 5XGK) was determined at a resolution of 2.80 Å and found to be similar to other* At*HPPD crystal structures (PDB ID: 1TFZ and 1TG5). The crystals belong to space group P 21 and comprise four molecules per asymmetric unit, which consists of two homodimers ([Supplementary-material supplementary-material-1] and [Supplementary-material supplementary-material-1]). Each monomeric unit consists of nine *α*-helices and sixteen *β*-strands that form two barrel-shaped structural domains: a conserved* C*-terminal domain containing the active site and a diversified N-terminal loop domain (Figure 1(a)). There is no reported catalytic function for the smaller* N*-terminal domain. The catalytic site and the metal ion are located in the C-terminal domain of HPPD, as mentioned in [30, 33, 38]. Electron density for a ligand chelating with the metal ion was only seen in molecular A ([Supplementary-material supplementary-material-1]). The structure of HPPA can be built into the density map (Figure 1(b)).

Extensive crystallographic and spectroscopic studies have shown that the metal ion is sixfold coordinated with the 2-His-1-Glu facial triad (His226 and His308 and one Glu394) and three water molecules at the active site [10, 30, 39]. Similarly, in HPPD-HPPA complex, the *α*-ketoglutarate moiety of HPPA in a bidentate interaction results in an octahedral coordination geometry involving the facial triad (Figure 1(b)). In addition to chelation with metal ion, the phenolic hydroxyl of HPPA forms a hydrogen bond with the side chain of Asn423 on the C-terminal *α*-helix (*α*9). A 75° horizontal rotation (Figure 1(c)), the benzene ring of HPPA shapes a T-*π* interaction with Phe381 and a weak hydrophobic interaction with the surrounding residues (such as Leu368 and Leu427, in [Supplementary-material supplementary-material-1]), leading to the current orientation of the aromatic moiety of HPPA. These interactions impose the formation of an edge-to-face conformer that is frequently found in biological systems. Apparently, the entrance of the active cavity is a hydrophobic surface and the internal surface is composed of a series of polar amino acids. Based on the alignment of the primary sequence of HPPD from various species, the facial triad and other amino acids involved in the interaction with the HPPA are highly conserved ([Supplementary-material supplementary-material-1]).

### 2.2. QM/MM Calculation

**Figure 2:**
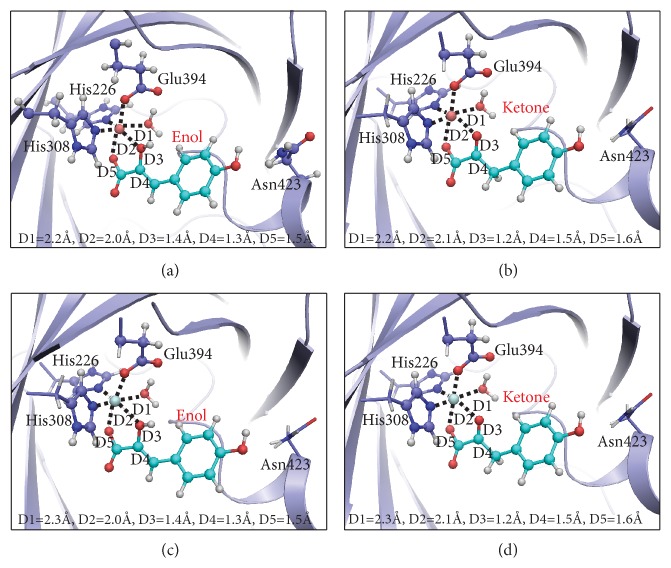
Optimized structures of HPPD complexed with different metal ions and isomers of HPPA: (a) Co^2+^ and enol; (b) Co^2+^ and ketone; (c) Fe^2+^ and enol; (d) Fe^2+^ and ketone. The geometries were optimized at the QM/MM (B3LYP/6-31+G*∗*: AMBER) level. The key distances in the figure are in angstroms. Cobalt, iron, carbon, oxygen, nitrogen, and hydrogen atoms are colored in pink, light green, cyan, red, blue, and white, respectively. The backbone of the protein is rendered in light blue. All QM atoms are represented as balls and sticks, and part of surrounding residues are rendered as sticks.

It is well-known that HPPA exists in the form of enol and ketone in aqueous solutions. Although the above crystal structure of HPPD-HPPA complex has been resolved, it is uncertain whether HPPA in the active site is enol or keto form, since hydrogen atoms cannot be determined in the crystal structure. Then, we performed QM/MM calculations to provide insight about the preferred tautomer bound to HPPD. Collected in Figure 2 are the QM/MM optimized geometries for HPPD complexed with different metal ions and isoforms of HPPD(Co^2+^)-HPPA(enol), HPPD(Co^2+^)-HPPA(ketone), HPPD(Fe^2+^)-HPPA(enol), and HPPD(Fe^2+^)-HPPA(ketone). Key interatomic distances and relative Gibbs free energies for these complexes are collected in Table 1. As can be seen from the table, all these structures are in good agreement with the experimental crystal structure with per atom RMSD ~0.38 Å, suggesting that these optimized geometries are reasonable. Among the parameters listed in Figure 2 and Table 1, D3 and D4 are those that distinguish between the enol and keto tautomers of HPPA. In the structure of HPPD (Co^2+^)-HPPA(enol), the optimized values for D3 and D4 are 1.42 Å and 1.33 Å, respectively, which are significantly different from D3 (1.24 Å) and D4 (1.54 Å) for HPPD (Co^2+^)-HPPA (ketone). In the crystal structure, the values of D3 and D4 are 1.19 Å and 1.53 Å, which are more consistent with the structure of HPPD(Co^2+^)-HPPA(ketone), suggesting that the HPPA is more likely to be a ketone in the crystal. This conclusion is further confirmed by the QM/MM free energy calculations, indicating that the HPPD(Co^2+^)-HPPA(ketone) is much more stable than the HPPD(Co^2+^)-HPPA(enol) by 19.26 kcal/mol (Table 1). A similar conclusion reached upon replacing Co^2+^ in the HPPD(Co^2+^)-HPPA(enol) and HPPD(Co^2+^)-HPPA(ketone) with Fe^2+^ where the keto structure is 13.27 kcal/mol more stable than the enol (Table 1). According to the above QM/MM calculation, it is likely that the metal and the existing form of HPPA in the active site of* At*HPPD-HPPA structure are Co^2+^ and keto form, respectively.

### 2.3. Structural Comparison of AtHPPD-HPPA Complex with Holo-AtHPPD

**Figure 3:**
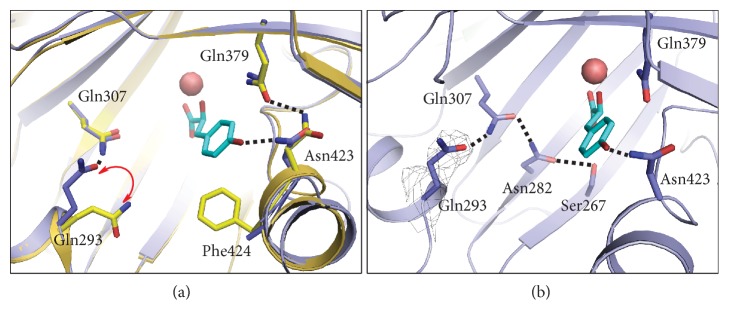
Structure comparison of* At*HPPD-HPPA complex with holo-*At*HPPD. (a) Superposition of the active sites of holo-*At*HPPD (yellow) and* At*HPPD-HPPA (light blue). (b) The H-bond network in the active site of* At*HPPD-HPPA complex. The key residues are shown as sticks and the H-bonds are indicated with black dash lines. Gray electron densities (2Fo–Fc map) correspond to Gln293 contoured at 1.0 *σ*.

To establish whether HPPD suffered from a conformational change upon the HPPA binding, we superimposed the holo-*At*HPPD structure (PDB ID: 1SQD) [30] with the structure of* At*HPPD-HPPA complex (Figure 3). As indicated in [Supplementary-material supplementary-material-1], a conformational alteration was observed for residue Phe428 on the* C*-terminal *α*-helix, which likely evades steric clashing with HPPA in the complex structure. Another difference is that the *β*-sheet fragment, Phe250-Phe253, rotated approximately 30°, and transformed to be a loop structure ([Supplementary-material supplementary-material-1]). HPPA forms an H-bond with Asn423 locating at the* C*-terminal *α*-helix (*α*9), while the H-bond between Gln379 and Asn423 presenting in holo-*At*HPPD structure disappeared in* At*HPPD-HPPA complex. It should be noted that the poor electron density for Phe424 residue in* At*HPPD-HPPA leads us to speculate that the Phe424 residue might be inherently flexible. Besides, Gln293 underwent a conformation change during the binding of HPPA (Figure 3(a)). More importantly, this conformation change made Gln293 involved in the formation of H-bond network of Ser267-Asn282-Gln307 which surrounds the HPPA in the active site (Figure 3(b)). As mentioned previously, those residues involved in the H-bonded network of Ser267-Asn282-Gln307 should have important roles in catalytic process [23, 25].

To further understand the roles of the residues involved in the aforementioned interactions, we performed site-directed mutagenesis of these residues and kinetically characterized those mutants. The* Michaelis-Menten* constants (*K*_m_) and the catalytic constants (*k*_cat_) were measured for wild type (WT)* At*HPPD and various mutants are shown in Table 2. Intriguingly, once the residue Asn423 that exhibits hydrogen bond interaction with HPPA was mutated to be Ala, the binding affinity against HPPA decreased by about 7-fold compared with that of wild type. As for the residue Phe381 that displaying T-*π* interaction with HPPA in* At*HPPD-HPPA complex, the affinity of F381A mutant against HPPA decreased about 5-fold. In addition, both of them resulted in 50% and 14% decrease in the catalytic rate constant (*k*_cat_), respectively. Hence, the results indicate that these two residues directly interacting with HPPA play important roles in the substrate binding but exhibit minor effects on the HPPD catalysis. In particular, the hydrogen bonding between Asn423 and HPPA may just provide an anchor to the following catalytic process.

Despite no direct interaction was observed between Gln293 and HPPA, Gln293 exhibited a conformation change upon the HPPA binding and subsequently involved in an important H-bond network surrounding the substrate (Figure 3(b)). To understand the role of Gln293 and the H-bond network, we mutated all of the corresponding residues. As depicted in Table 2, all of these mutants show sharp decreases in *k*_cat_ values when comparing with that of WT which indicates that they are highly involved in the underlying catalytic process, although none of them are directly involved in substrate binding process. Among them, the mutant Q293A possesses both the most remarkable decrease (about 76-fold) in *k*_cat_ and the greatest attenuation (about 47-fold) in binding affinity (*K*_m_) with HPPD, which demonstrates its critical role in the substrate binding and the following catalytic conversion.

Taken together, we can conclude that the HPPD undergoes significant conformational changes upon HPPA binding, in which the rotation of residue Gln293 may be regarded as the most prominent hallmark. In more detail, Gln293 must undergo a conformational change that benefits to the formation of the H-bond network of Ser267-Asn282-Gln307-Gln293. For convenience, we define the holo-*At*HPPD as inactive state and the HPPA-bound* At*HPPD as active state. On the basis of the structural comparison, the remarkable characteristic of inactive state is the H-bond network formed by Glu394-Gln379-Asn423, whereas the key feature for the active state is the formation of the H-bond network by Ser267-Asn282-Gln307-Gln293. Such difference of H-bond network between inactive state and active state is mainly attributed to the conformational change of residue Gln293 and Asn423. In other words, Gln293 must undergo a significant rotation to form H-bond with Gln307, which triggers the generation of the H-bond network of Ser267-Asn282-Gln307-Gln293 to stabilize the conformation of HPPA in the catalytic pocket. After the binding of HPPA, the formation of H-bond between Asn423 and the hydroxyl group of HPPA led to the disappearance of the H-bond between Gln379 and Asn423 that exhibits in holo structure, further resulting in the breakdown of H-bond network of Glu394-Gln379-Asn423. After combining the above analysis, we believe that blocking the conformational transition of Gln293 between inactive and active states should be an effective way to design new inhibitors.

### 2.4. Structure-Based Herbicide Design

**Figure 4:**
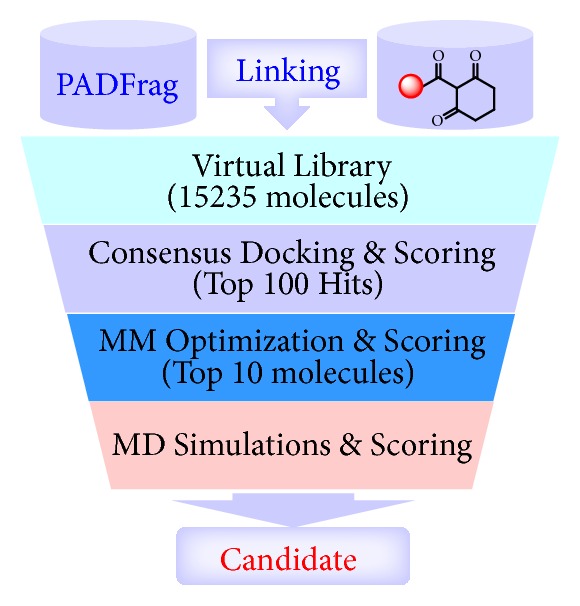
Computational protocol of discovering new HPPD inhibitors.

As aforementioned, upon the binding of HPPA, Gln293 must undergo a significant conformational conversion from inactive to active state prior to the enzymatic catalysis. Blocking such a conformational change of Gln293 should be an effective way to design new inhibitors. Therefore, we proposed a computational protocol (Figure 4) to discover new HPPD inhibitors based on the inactive state of HPPD. First, we constructed a virtual library containing 15,235 compounds by linking the fragments in PADFrag [40] with the cyclohexane-1,3-dione as pharmacophore. Then, a consensus docking strategy was adapted to carry out virtual screening towards the virtual library based on the structure of inactive state of HPPD. This step produced 100 hit molecules ([Supplementary-material supplementary-material-1]) according to the ranking results of the scoring functions. Subsequently, molecular mechanics optimization was carried out for the selected 100 docking models and the binding energies of each compound were recalculated ([Supplementary-material supplementary-material-1]). Then, 10 compounds with the most favorable binding energies ([Supplementary-material supplementary-material-1] and [Supplementary-material supplementary-material-1]) were subjected to further molecular dynamic simulations using Amber16 program [41]. Finally, the compound with the most favorable binding free energy was selected for further synthesis and bioassay.

**Figure 5:**
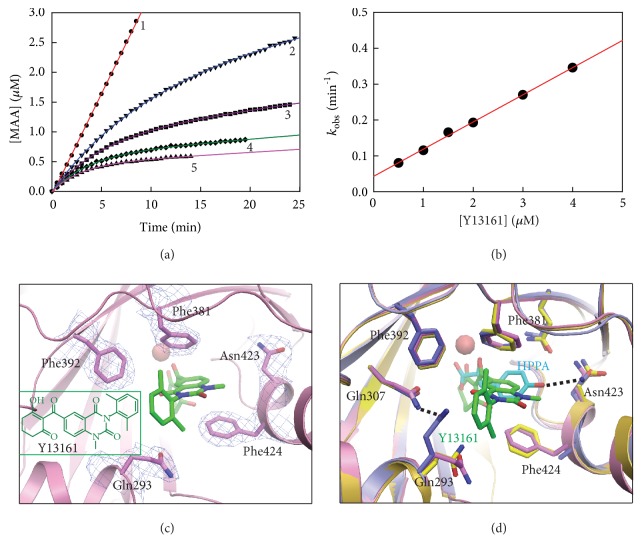
Inhibitory kinetics of* At*HPPD by compound Y13161 and structural analysis of* At*HPPD-Y13161 complex. (a) Progress curves in the presence of varying concentrations of Y13161 on the inhibition of* At*HPPD. The assays were carried out in the presence of 80 *μ*M HPPA and various concentrations of compound Y13161 (1, 0 *μ*M; 2, 1.0 *μ*M; 3, 2.0 *μ*M; 4, 3.0 *μ*M; and 5, 4.0 *μ*M). (b) Secondary plot of *k*_obs_ against concentrations of compound Y13161. Experimental data are shown as solid black dots and fitting lines as colored solid lines. (c) The close-up view of the active site illustrating the binding mode of Y13161 (shown in green stick). Light blue electron densities (2Fo–Fc map) correspond to the key residues contoured at 1.0 *σ*. (d) The superposition of the active sites of* At*HPPD-Y13161 complex (pink) with holo-*At*HPPD (yellow) and* At*HPPD-HPPA complex (light blue, HPPA showed as cyan stick). The key residues are shown as sticks and H-bond interactions are shown as dash lines.

Based on the computational protocol in Figure 4, a new compound (experimental code: Y13161) was eventually discovered as a new potent HPPD inhibitor. Y13161 was prepared by an eight-step synthetic route ([Supplementary-material supplementary-material-1]) by using 5-methyl-2-nitrobenzoic acid as starting material, and its structure was confirmed by ^1^H NMR, ^13^C NMR and HRMS spectral data (Figures [Supplementary-material supplementary-material-1]–[Supplementary-material supplementary-material-1]). Inhibitory kinetic analysis towards the recombinant* At*HPPD indicated that Y13161 is a competitive inhibitor (*K*_i_ = 24.16 ± 1.01 nM) with respect to HPPA with the characteristic of slow binding (Figures 5(a), 5(b), and [Supplementary-material supplementary-material-1]). It is also found that Y13161 shows much lower potency towards human HPPD (*K*_i_ = 179.0 ± 7.70 nM, [Supplementary-material supplementary-material-1]), which means that the compound might be low toxic to humans.

To validate the applicability of the molecular design strategy that shown in Figure 5, we determined the crystal structure of* At*HPPD in complex with Y13161 (PDB ID: 5YY6) and compared the structure of* At*HPPD-Y13161 with the holo-*At*HPPD and the* At*HPPD-HPPA complex, respectively. As expected, the binding mode of Y13161 in this crystal structure is very similar to that of the MD simulated model ([Supplementary-material supplementary-material-1]). Similar to all the known inhibitors, two carbonyl groups on Y13161 formed clear chelation interaction between its two carbonyl groups with the metal ion and a pronounced *π*-*π* stacking interaction between quinazoline-2,4-dione moiety and two aromatic residues (Phe381 and Phe424) (Figures 5(c), [Supplementary-material supplementary-material-1], and [Supplementary-material supplementary-material-1]). Additionally, the cyclohexane moiety interacts with the surrounding hydrophobic residues like Phe419, Pro280, Val269, and Val228 ([Supplementary-material supplementary-material-1]). Intriguingly, the benzene ring at the N-1 position of the quinazoline-2,4-dione ring formed *π*-*π* stacking interaction with Phe392 (Figure 5(c)), which results in the more complementary in shape to HPPD active site. It should be pointed out that the interaction with Phe392 has never been observed before in other HPPD inhibitor complexes. Most importantly, the conformation of the active site of* At*HPPD-Y13161 overlaps very well with that of the holo-*At*HPPD, but remarkably different from that of* At*HPPD-HPPA complex, suggesting that the binding of Y13161 indeed keep HPPD in inactive state as expected (Figure 5(d)). As a consequence, the H-bond network of Ser267-Asn282-Gln307-Gln293 would not form due to the far distance (~5.0 Å) between Gln293 and Gln307. On the contrary, the H-bond network of Glu394-Gln379-Asn423 appears around the Y13161, just like its presence in the active site of holo-*At*HPPD structure. Collectively, these observations confirmed solidly that targeting the conformational change of Gln293 is an effective way to design new HPPD inhibitors.

Very fortunately, further extensive greenhouse and field trial showed that Y13161 exhibits excellent herbicidal activity against most of broadleaf weeds and some important grasses at the application dosage of 100~150 g.ai/ha ([Supplementary-material supplementary-material-1]). In addition, Y13161 showed excellent crop safety against sorghum even at the dosage as high as 600 g.ai/ha ([Supplementary-material supplementary-material-1]). To our knowledge, there is so far no commercial postemergence selective herbicide for sorghum weed control, although it is the fifth largest cereal crop in the world with a planting area of about 50 million hectares. In addition, the LD_50_ of Y13161 for male and female rats was both over 5000 mg/kg. Moreover, no critical eye and skin irritation or skin sensitization was recognized on both active ingredient and formulation. Prolonged exposure studies demonstrated that Y13161 exhibited no evidence of carcinogenicity, teratogenicity, and reproduction toxicity. Toxicity of Y13161 towards nontarget aqueous species (fish, crustaceans, and algae), honeybees, and birds was also quite low. Overall, due to its excellent herbicidal activity, crop safety, and toxicological properties, Y13161 (the approved common name is* Benquitrione*) has progressed into industry development and will get registration as a selective herbicide for the weed control in sorghum field from the Institute for the Control of Agrochemicals, Ministry of Agriculture (ICAMA) of China.

## 3. Discussion

In this work, we successfully resolved the crystal structure of* At*HPPD complexed with its natural substrate HPPA for the first time. Then, by combining with QM/MM calculation, we revealed that the keto rather than enol tautomer is the real existing form of HPPA in the binding pocket. Subsequently, Ser267, Asn282, Gln307, Asn423, and Gln293 were identified as the key residues playing significant roles in the HPPD catalysis. Finally, we proposed a new molecular design strategy of blocking the conformational switch of Gln293 from the inactive to active state and accordingly leading to the discovery of a new herbicide for the weed control in sorghum field.

HPPD has been identified as an important target for drug and herbicide discovery. To our knowledge, all the known HPPD inhibitors are competitive with respect to the substrate; however, the exact binding mode of HPPA with HPPD at atomic level remains unavailable. Due to the critical importance of the binding mode of HPPA, three major HPPD-HPPA interaction models [25] ([Supplementary-material supplementary-material-1]) have been proposed based on the crystal structure of HPPD in complex with acetate [32], inhibitors [31] or the crystal structure of product bound HMS [35], and an HPPD homologue with about 40% sequence similarity and share the same substrate. These models endorse the bidentate coordination between the a-keto acid moiety of HPPA with the metal ion and a conserved hydrogen bonding with a glutamine residue. However, the orientation of the benzene ring of HPPA is significantly different from each other, resulting in that the 4-hydroxyl group of HPPA interacted with different residues. The crystal structure of* At*HPPD-HPPA showed clearly that the 4-hydroxyl group of HPPA formed H-bond with Asn423 rather than Ser267 or Gln307. The a-keto acid moiety of HPPA only formed the conserved bidentate coordination with metal ion. The hydrogen bond interaction between the carbonyl oxygen of the a-keto acid moiety and Gln379 proposed by Serre et al. [32] and Harrison et al. [35] was not observed in the crystal structure of* At*HPPD-HPPA. Of course, this does not rule out the possibility that Gln379 plays an important role in the catalytic cycle.

It is well-known that the study of enzymatic reaction mechanism by QM/MM calculations is critically dependent on the starting conformation of substrate. A recent QM/MM study performed by Wójcik et al. [23] obtained the HPPD-Fe(IV)=O-HPA complex by MD simulation. The MD model was prepared on the basis of the crystal structure of* pseudomonas fluorescens* HPPD (PDB code: 1CJX) in which the ligand acetate was replaced by HPA (4-hydroxyphenylacetate) and the ferrous ion was substituted with an iron-oxo group. The authors evaluated the reasonability of this modeled complex based on the crystal structure of product bound HMS (PDB code: 2R5V), as well as the previous biochemical studies. Several residues (Ser267, Asn282, Gln293, and Gln307) were identified to play vital roles in HPPD catalysis. Our study also provides additional evidences that S267A, N282A, Q307A, and Q293A mutations decreased the catalytic efficiency by ~12-fold, ~160-fold, ~99-fold, and ~3722-fold, respectively. The QM/MM in this study enhanced the understanding on the HPPD catalytic reaction pathway, especially on the two key steps of ring hydroxylation and the carboxymethyl migration. However, in the current study, the structural information of HPPD-HPPA complex clearly demonstrates the direct H-bonding interaction of HPPA with Asn423, which was never noticed before. Moreover, the N423A mutation significantly reduced the catalytic efficiency, which further confirmed the importance of Asn423. Therefore, Asn423 should be paid much attention in the future study of enzymatic reaction mechanism.

Due to the successful determination of the crystal structure of HPPA-bound* At*HPPD for the first time, it is possible for us to compare the structural difference between HPPA-bound* At*HPPD and holo-*At*HPPD. As a result, the conformational change of Gln293 was observed upon the HPPA binding. Then, we defined two states of the enzyme, inactive state, and active state, according to the conformational characteristics of Gln293. In order to bind HPPA, Gln293 must undergo a conformational change from inactive state to active state so that the H-bond network of Ser267-Asn282-Gln307-Gln293 can be established to stabilize the conformation of HPPA in the catalytic center. In other words, without the formation of the H-bond network, the binding of HPPA is so unstable that the enzymatic reaction will not occur. Inspired by this observation, we proposed a new molecular design strategy for the first time by taking the conformational change of Gln293 as a target and successfully designed a novel nanomolar inhibitor of* At*HPPD, which has progressed into industry development as the first selective herbicide for the weed control in sorghum field.

## 4. Materials and Methods

### 4.1. Protein Expression and Purification

The* pET15b*-*At*HPPD plasmid containing an* N*-terminal His tag and the full-length* At*HPPD protein (UniProtKB-P93836) was used as described previously [13, 14, 16, 42]. Mutants were prepared by using the Quickchange™ Site-directed Mutagenesis Kit (Stratagene) and confirmed by sequencing. The recombinants were induced with 0.2 mM IPTG and expressed in* E. coli* BL21 (DE3) for 14 h at 18°C. Overexpressed recombinants were lysed in buffer that containing 20 mM HEPES, pH 7.5 and 150 mM NaCl. The supernatant was purified by affinity chromatography (Ni-NTA agarose, Qiagen) and ion exchange chromatography (Source 15Q, GE Healthcare) under standard conditions. Protein analysis by using a Superdex 200 increases 10/300 GL column (GE Healthcare) run at 0.5 mL/min in buffer (10 mM Tris·HCl, pH 7.3, 50 mM KCl). Enzyme was stored at −80°C and the concentration was determined by using BCA Protein Assay Kit (Biosharp).

### 4.2. Activity Assay and Inhibition Kinetics

According to the reported coupled assay [15, 16, 43, 44], the catalytic activity of* At*HPPD was determined in 1.8 mL of reaction mixture containing 20 mM HEPES (pH 7.0), 0.1 mM FeSO_4_, 2 mM sodium ascorbate, an appropriate amount of HPPA, sufficient homogentisate 1, 2-dioxygenase (HGD) and 14 nM* At*HPPD at 25°C. The reaction was initiated by enzyme and detected absorbance change at 318 nm [30, 45] by using a spectrophotometer (Perkin-Elmer Lambda 45) equipped with a magnetic stirrer in the cuvette holder. For inhibitory kinetic studies, what should be noticed is that the compound tested here is slow binding, competitive inhibitor. The reaction mechanism and equations (see details in [Supplementary-material supplementary-material-1]) used here according to the reports [43, 44]:

(1) $$\begin{array}{c} \left[\;P\;\right]=v_{s}t+\frac{v_{0}-v_{s}}{k_{obs}}\left(\;1-e^{-k_{obs}t}\;\right) \end{array}$$

where *v*_0_ and *v*_s_ are the initial and steady-state velocities of the reaction in the presence of inhibitor, and *k*_obs_ is the observed first order rate constant. Inhibitor was dissolved in dimethyl sulfoxide (DMSO) to prepare a stock solution, from which various concentrations were prepared with reaction buffer just before use.

### 4.3. Crystallization and Structure Determination

The initial complex of HPPD with metal ion was prepared by adding 0.6 mM CoCl_2_ into the purified HPPD (0.3 mM). Next, the substrate (HPPA) was added to the above mixture with a final concentration of 1.0 mM and compound Y13161 was added with a final concentration of 0.5 mM. Crystals were obtained using the hanging drop vapor diffusion method after extensive optimization of the conditions. The reservoir solution consisted of 36% (w/v) polyethylene glycol 400, 0.1 M NaCl, and 0.1 M sodium acetate buffer, pH 5.0 for* At*HPPD-HPPA. In order to get the crystals with better diffraction quality, we cloned an N-terminal truncated form of* At*HPPD (residues Val33-Trp393) into the pPH expression vector. The method of expression and purification is the same as mentioned above. The reservoir solution of* At*HPPD-Y13161 consisted of 0.09 M Halogens (0.3 M Sodium fluoride, 0.3 M Sodium Bromide and 0.3 M Sodium iodide), 0.1 M Buffer System 3 pH 8.5 (1.0 M Tris(base) and 1.0 M Bicine), and 50% Precipitant Mix4 (25% v/v MPD, 25% PEG1000 and 25% PEG3350). Crystals were incubated at 18°C and suitable for diffraction studies in about one week.

The diffraction datasets were collected at beamline 17U (*At*HPPD-HPPA) and 19U (*At*HPPD-Y13161) at Shanghai Synchrotron Radiation Facility and processed with the HKL2000 suite [46]. The crystals of* At*HPPD-HPPA belong to space group P 21 and contain four molecules per asymmetric unit (two dimers), while* At*HPPD-Y13161 crystals belong to space group C 1 2 1 and there is only one monomer in an asymmetric unit. These structures were subsequently solved by molecular replacement using Phaser [47] with* At*HPPD bound to NTBC (PDB: 1TFZ) as the search model. The standard refinement was performed with programs PHENIX [48] and Coot [49] (final statistics for the structure are given in [Supplementary-material supplementary-material-1]). All structural representations in this paper were prepared with PYMOL (http://www.pymol.org).

### 4.4. QM-MM Calculation

The initial structure of HPPD complexed with the enol form of HPPA and with the Co^2+^ ion was constructed from the crystal structure of* At*HPPD-HPPA obtained in this work. The missing hydrogen atoms was added by using the Leap program implemented in AMBER16 package [41]. For the sake of convenience, this structure was named as HPPD(Co^2+^)-HPPA(enol). The constructed Michaelis-Menten complex structure was then subjected to a 500-step minimization simulation in which all heavy atoms were fixed. The minimized structure was used to prepare the pseudo-bond first-principles QM/MM calculations. The QM region was described in Figure 2 and the rest part of the structure was defined as MM region. Prior to the QM/MM geometry optimizations, the HPPD(Co^2+^)-HPPA(enol) structure obtained in previous step was energy-minimized with the MM method by using our revised version of the AMBER16 program, where the convergence criterion is a root-mean-square deviation (RMSD) of the energy gradient of less than 0.1 kcal·mol^−1^·Å^−1^. With an iterative energy minimization procedure, the minimized structures were optimized by the pseudo-bond QM/MM calculations at the B3LYP/6-31+G*∗*: AMBER level, in which the QM calculations were performed at the B3LYP/6-31+G*∗* level of theory by using a modified version of the Gaussian09 program [50], and the MM calculations were performed by using a modified version of the AMBER16 program. Frequency analyses were performed to characterize the optimized structures and to obtain the thermodynamic parameters of HPPD(Co^2+^)-HPPA(enol).

The QM/MM optimized HPPD(Co^2+^)-HPPA(enol) structure was used for the preparation of the initial structures of three other complexes, namely, HPPD(Co^2+^)-HPPA(ketone), HPPD(Fe^2+^)-HPPA(enol) and HPPD(Fe^2+^)-HPPA(ketone). The HPPD(Co^2+^)-HPPA(ketone) structure was prepared by detaching the hydrogen atom from the hydroxyl group of the enol moiety and reattaching it to the carbon atoms. The HPPD(Fe^2+^)-HPPA(enol) and HPPD(Fe^2+^)-HPPA(ketone) structures were prepared by, respectively, replacing the Co^2+^ ions in the optimized HPPD(Co^2+^)-HPPA(enol) and as prepared HPPD(Co^2+^)-HPPA(ketone) structures with Fe^2+^ ions. The constructed three complex structures were then subjected to QM/MM geometrical optimization followed by normal mode analyses. Assuming that the entropy contribution from MM region was largely cancelled out, the total Gibbs free energy of each complex structure was taken as the corresponding QM/MM energy and thermodynamic corrections to Gibbs free energy for the QM region.

### 4.5. Computational Simulation

For the structure-based herbicide design, the computational protocol is just as described previously. The detailed methods and results of structure-based virtual screening, structure optimization, and MD simulation can be seen in the Supplementary Information [Supplementary-material supplementary-material-1], Tables [Supplementary-material supplementary-material-1]–[Supplementary-material supplementary-material-1], and Figures [Supplementary-material supplementary-material-1] and [Supplementary-material supplementary-material-1].

## Figures and Tables

**Table 1:** Key interatomic distances (in Å) and relative Gibbs free energies (in kcal/mol) for different HPPD complexes.

Complex^*∗*^	Interatomic distances					Grel	RMSD ^†^
	D1	D2	D3	D4	D5		
HPPD(Co^2+^)-HPPA(enol)	2.210	2.032	1.421	1.334	1.523	0.00	0.141
HPPD(Co^2+^)-HPPA(ketone)	2.218	2.134	1.243	1.541	1.579	-19.26 ^‡^	0.043
HPPD(Fe^2+^)-HPPA(enol)	2.276	2.021	2.392	1.321	1.510	0.0	0.551
HPPD(Fe^2+^)-HPPA(ketone)	2.317	2.142	1.220	1.512	1.574	-13.27 ^§^	0.073
Exp.	2.171	2.098	1.185	1.529	1.530		0.000

^*∗*^ The naming of complexes and interatomic distances are described in Figure 2.

^†^ The RMSD (in Å) of the calculated distances (D1 to D5) from those in the X-ray crystal structure.

^‡^ Calculated by using the HPPD(Co^2+^)-HPPA(enol) complex as reference.

^§^ Calculated by using the HPPD(Fe^2+^)-HPPA(enol) complex as reference.

**Table 2:** Comparison of apparent kinetic parameters for reaction of the WT and mutants of *At*HPPD at pH 7.0^*∗*^. Each experiment was carried out in triplicate.

Enzyme	*k*_cat_ (s^−1^)	*K*_m_ (*μ*M)	*k*_cat_/*K*_m_
WT	1.079 ± 0.053	1.87 ± 0.37	0.5770 ± 0.1142
N423A	0.545 ± 0.061	15.66 ± 4.29	0.0348 ± 0.0095
F381A	0.924 ± 0.032	9.10 ± 0.94	0.1015 ± 0.0105
Q293A	0.014 ± 0.001	90.41 ± 11.33	0.00015 ± 0.00001
Q307A	0.176 ± 0.004	30.31 ± 1.67	0.0058 ± 0.0003
S267A	0.249 ± 0.025	5.33 ± 1.59	0.0467 ± 0.0139
N282A	0.039 ± 0.003	10.83 ± 2.33	0.0036 ± 0.0008

^*∗*^ Reactions were conducted in air-saturated 20 mM HEPES (pH 7.0) at 25°C and kinetically analyzed as described in Experimental Procedures. Errors for individual parameters were obtained from the nonlinear regression fit of the data to the Michaelis-Menten equation.

## Data Availability

Coordinates and structure factors have been deposited in the Protein Data Bank under accession codes of 5XGK (*At*HPPD-HPPA) and 5YY6 (*At*HPPD-Y13161).

## Supplementary Materials

Supplementary Materials Figure S1. Three proposed binding models of HPPA in HPPD active site. Figure S2. Gel filtration analyses to analyze the oligomerization state of the purified* At*HPPD and the SDS-PAGE of* At*HPPD. Figure S3. Overall structure of* At*HPPD-HPPA complex. Figure S4. Overall binding mode of HPPA in* At*HPPD active site. Figure S5. Sequence alignment of HPPD from different species. Figure S6. Structural comparison of* At*HPPD-HPPA complex with holo-*At*HPPD structure. Figure S7. Time dependence of the RMSD of protein backbone atoms and ten candidates during the MD simulation. Figure S8. ^1^H NMR spectral of Y13161 in CDCl_3_. Figure S9. ^13^C NMR spectral of Y13161 in CDCl_3_. Figure S10. HRMS spectrum of Y13161. Figure S11. Inhibitory kinetics of* At*HPPD by compound Y13161. Figure S12. Inhibitory kinetics of* h*HPPD by compound Y13161. Figure S13. Comparison of docking binding mode and MD simulated model with cocrystal structure of* At*HPPD- Y13161. Figure S14. The interactions of Y13161 with* At*HPPD. Table S1. Data collection and refinement statistics for the* At*HPPD-HPPA and* At*HPPD-Y13161 complex. Table S2. Top 100 compounds from virtual screening with their rank. Table S3. Binding free energy evaluation (kcal/mol) for the top 100 compounds after the structure optimization. Table S4. Binding free energy (kcal/mol) calculated for top 10 compounds after structure minimization and MD simulation. Table S5. Herbicidal activity of Y13161 and Mesotrione. Table S6. Crop selectivity of Y13161 and Mesotrione (150 g ai/ha). Method S1. Preparation of compound Y13161. Method S2. Inhibitory kinetics of HPPD. Method S3. Computational simulation.
