# Supplementary material for: Crystal Structure of 4-Hydroxyphenylpyruvate Dioxygenase in Complex with Substrate Reveals a New Starting Point for Herbicide Discovery
Source: Research (Wash D C). 2019 Jul 8;2019:2602414. doi: 10.34133/2019/2602414 (PMC6750108; doi:10.34133/2019/2602414)
Supplement: Supplementary Materials — Figure S1. Three proposed binding models of HPPA in HPPD active site. Figure S2. Gel filtration analyses to analyze the oligomerization state of the purified AtHPPD and the SDS-PAGE of AtHPPD. Figure S3. Overall structure of AtHPPD-HPPA complex. Figure S4. Overall binding mode of HPPA in AtHPPD active site. Figure S5. Sequence alignment of HPPD from different species. Figure S6. Structural comparison of AtHPPD-HPPA complex with holo-AtHPPD structure. Figure S7. Time dependence of the RMSD of protein backbone atoms and ten candidates during the MD simulation. Figure S8. 1H NMR spectral of Y13161 in CDCl3. Figure S9. 13C NMR spectral of Y13161 in CDCl3. Figure S10. HRMS spectrum of Y13161. Figure S11. Inhibitory kinetics of AtHPPD by compound Y13161. Figure S12. Inhibitory kinetics of hHPPD by compound Y13161. Figure S13. Comparison of docking binding mode and MD simulated model with cocrystal structure of AtHPPD- Y13161. Figure S14. The interactions of Y13161 with AtHPPD. Table S1. Data collection and refinement statistics for the AtHPPD-HPPA and AtHPPD-Y13161 complex. Table S2. Top 100 compounds from virtual screening with their rank. Table S3. Binding free energy evaluation (kcal/mol) for the top 100 compounds after the structure optimization. Table S4. Binding free energy (kcal/mol) calculated for top 10 compounds after structure minimization and MD simulation. Table S5. Herbicidal activity of Y13161 and Mesotrione. Table S6. Crop selectivity of Y13161 and Mesotrione (150 g ai/ha). Method S1. Preparation of compound Y13161. Method S2. Inhibitory kinetics of HPPD. Method S3. Computational simulation. [file 2602414.f1.pdf]

Supporting Information

Crystal Structure of 4-Hydroxyphenylpyruvate Dioxygenase in Complex with Its Natural Substrate Reveals A New Starting Point for Herbicide Discovery

Hong-Yan Lin,[1,3 †] Xi Chen,[2†] Jia-Nan Chen,[1] Da-Wei Wang,[1] Feng-Xu Wu,[1] Song-Yun Lin,[3] Chang-Guo Zhan,[1] Jia-Wei Wu,[3] Wen-Chao Yang,[1*] Guang-Fu Yang[1,4 *]

[1]Key Laboratory of Pesticide & Chemical Biology of Ministry of Education, International Joint Research Center for Intelligent Biosensor Technology and Health, College of Chemistry, Chemical Biology Center, Central China Normal University, Wuhan 430079, P.R. China; [2]College of Chemistry and Material Science, South-Central University for Nationalities, Wuhan 430074, P. R. China; [3]MOE Key Laboratory of Protein Sciences, Tsinghua-Peking Center for Life Sciences, School of Life Sciences, Tsinghua University, Beijing 100084, P. R. China; [4]Collaborative Innovation Center of Chemical Science and Engineering, Tianjin 30071, P.R. China.

[†] These two authors made equal contributions.

[*]Wen-Chao Yang, e-mail: tomyang@mail.ccnu.edu.cn; Tel: 86-27-67867706; Fax: 86-27-67867141.

[*]Guang-Fu Yang, e-mail: gfyang@mail.ccnu.edu.cn; Tel: 86-27-67867800; Fax: 86-27-67867141.

Supplemental Figures

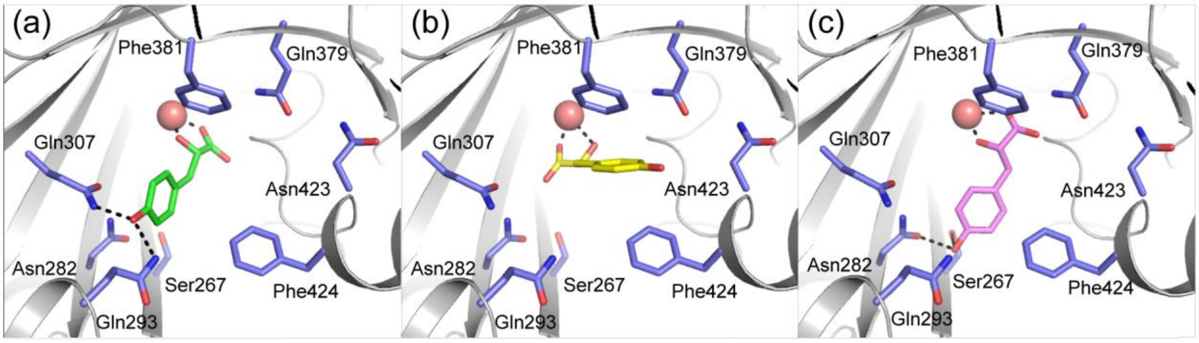

Figure S1 Three proposed binding models of HPPA in HPPD active site. (a) Inspired by the crystal structure of *pseudomonas fluorescens* HPPD. (b) Hypothesized according to the crystal structure of *streptomyces avermitilis* HPPD complexed with NTBC. (c) Hypothesized according to the HMA binding mode in hydroxymandelate synthase. The key residues showed as purple sticks, and HPPA showed as green, yellow and pink sticks, respectively.

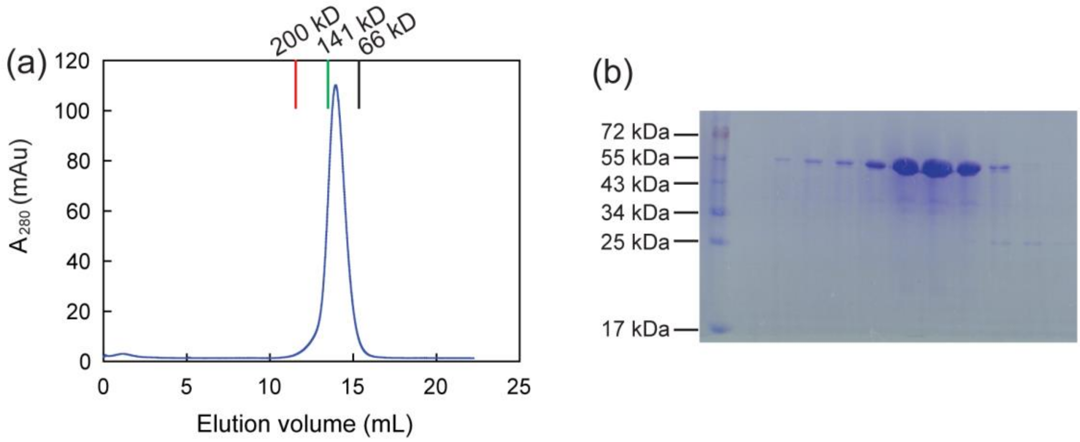

Figure S2 Gel filtration analyses to analyze the oligomerization state of the purified *At*HPPD (a) and the SDS-PAGE of *At*HPPD (b).

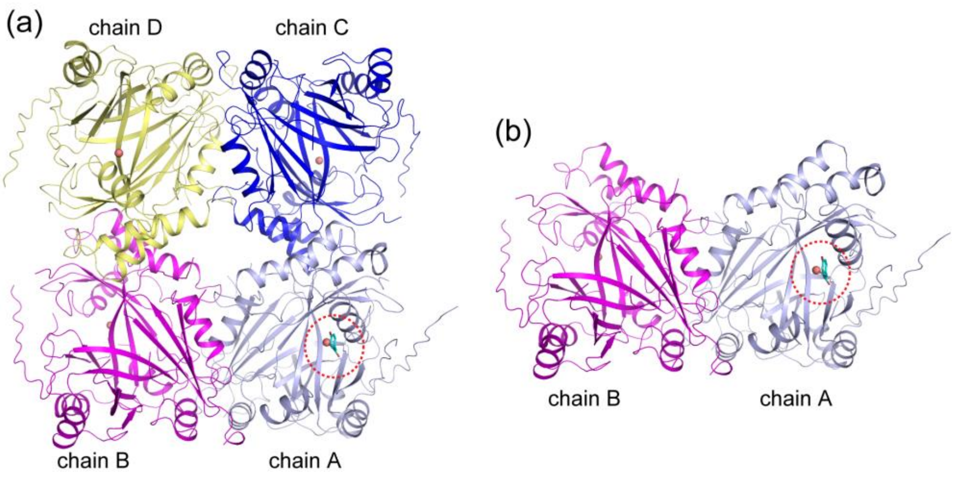

Figure S3 Overall structure of *At*HPPD-HPPA complex. (a) Four molecules in one asymmetric unit which is made up of two homodimers. The metal ion (M) in the active site is shown as deep salmon sphere. HPPA was shown in cyan stick. (b) Ribbon diagram of homodimer structure.

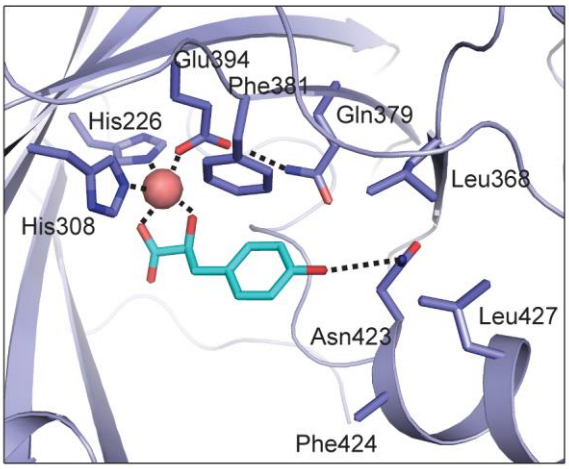

Figure S4 Overall binding mode of HPPA in *At*HPPD active site. HPPA forms T-π interaction with Phe381 and weak hydrophobic interaction with residues Leu368 and Leu427.

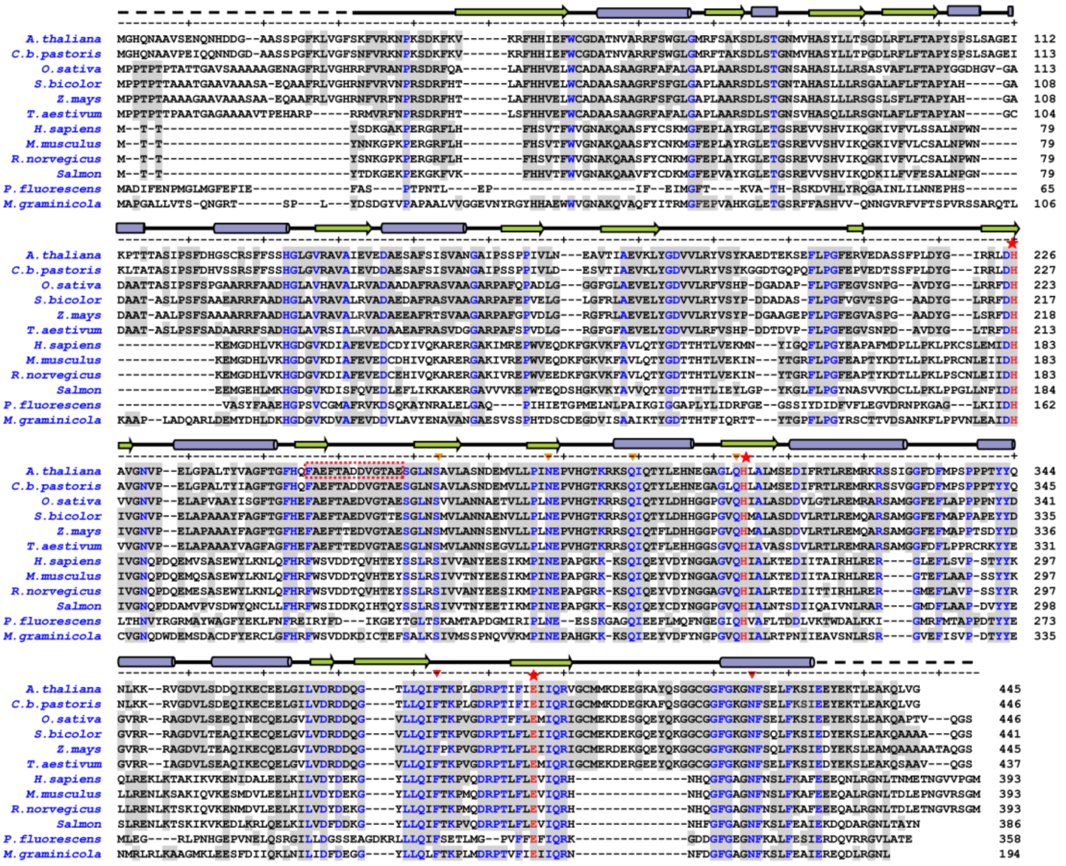

Figure S5 Sequence alignment of HPPD from different species. The facial triad residues involved in the chelation with Fe^{2+} are shown in red and indicated by red stars. Residues involved in the direct interactions with HPPA indicated by red triangle, while those involved in the H bond network indicated by orange triangle. Other conserved residues are shown in blue.

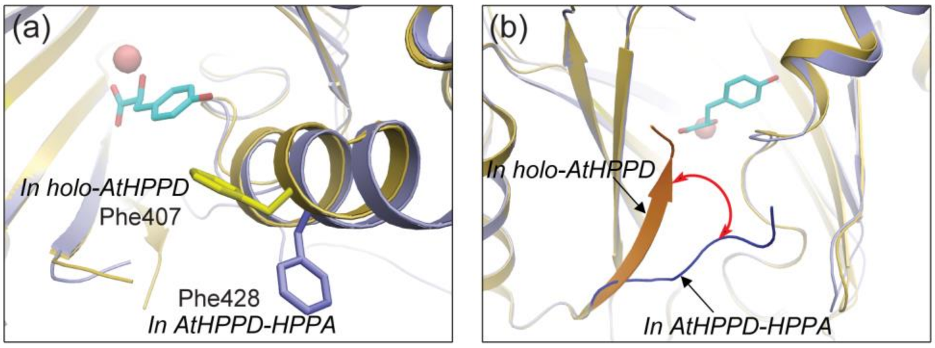

Figure S6 Structural comparison of *At*HPPD-HPPA complex (light blue) with holo-*At*HPPD structure (yellow). (a) The conformational alteration of residue Phe428 on the *C*-terminal α-helix. (b) The β-trand fragment (framed with red line in figure S5) rotated about 30° and

transformed to be a loop structure.

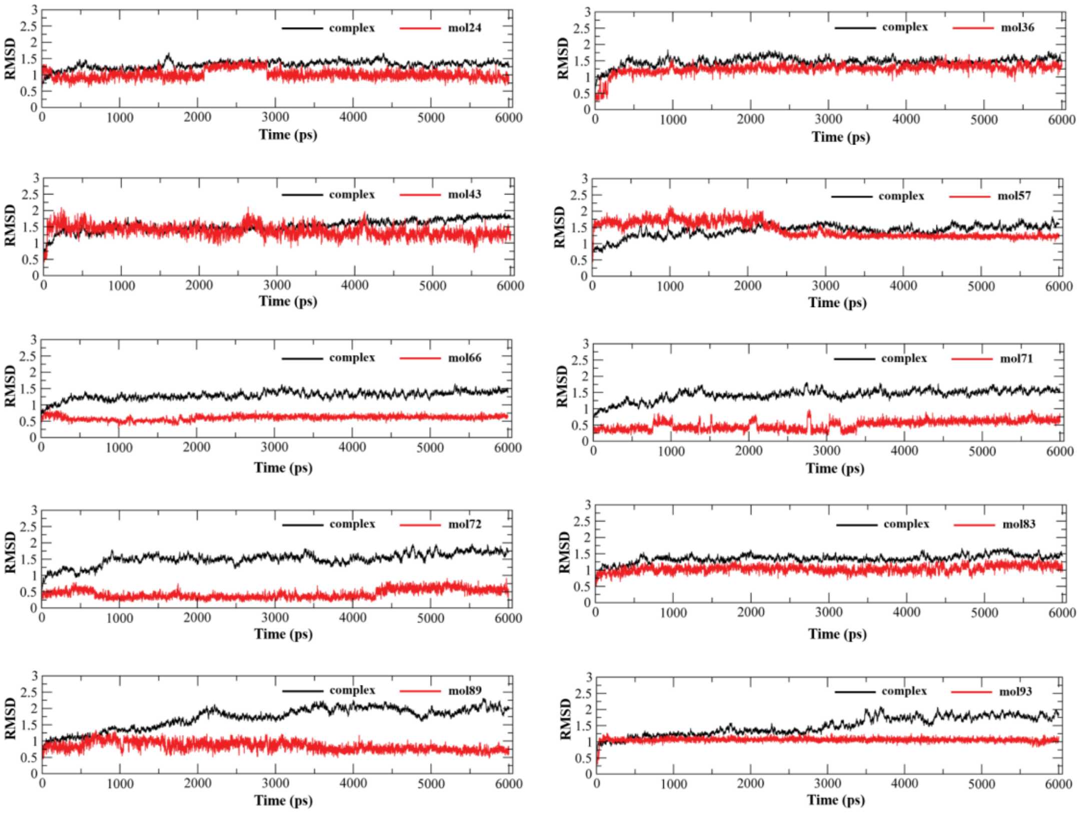

Figure S7 Time dependence of the RMSD of protein backbone atoms (color in black) and ten candidates (color in red) during the MD simulation.

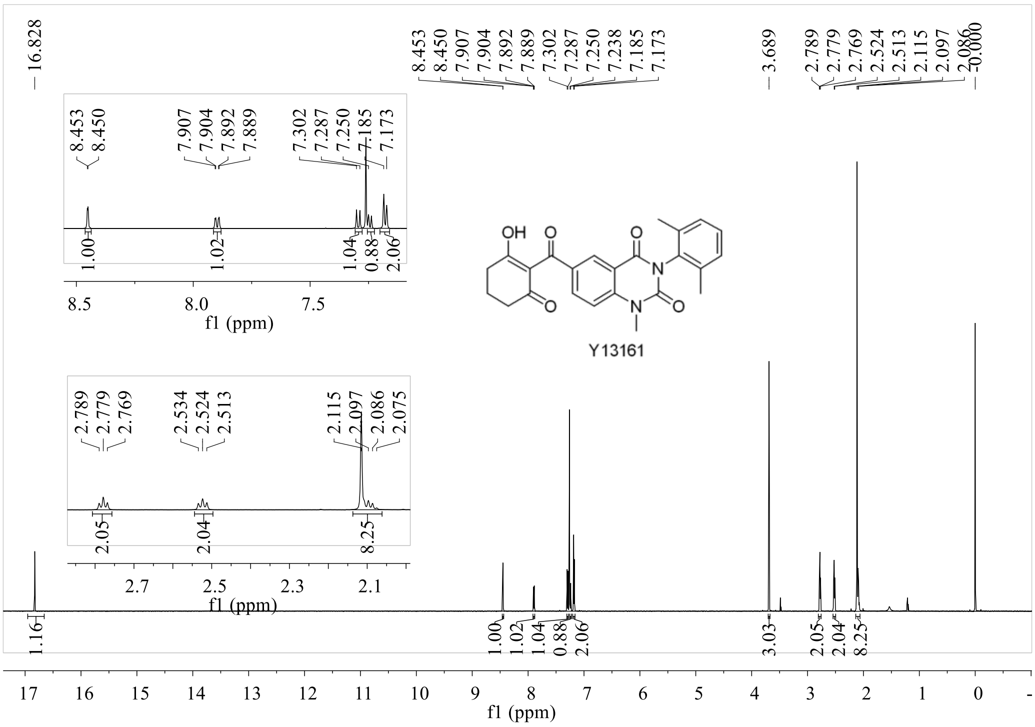

Figure S8 ^{1}H NMR spectral of **Y13161** in $CDCl_3$.

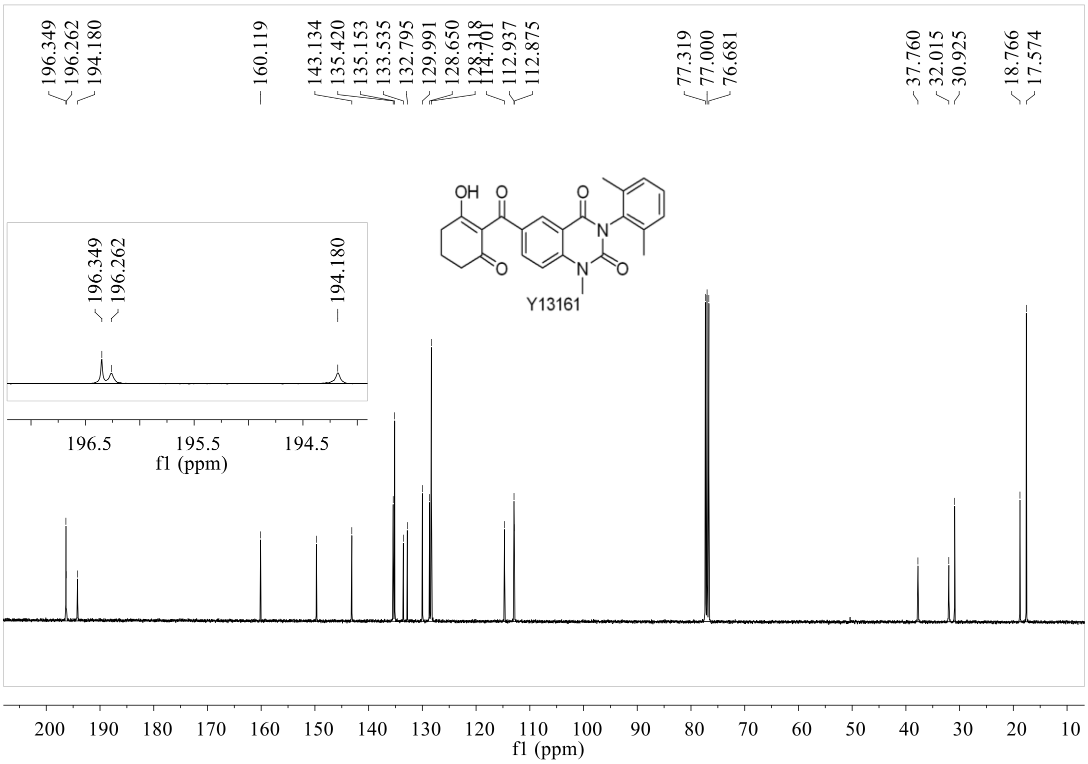

Figure S9 ^{13}C NMR spectral of **Y13161** in $CDCl_3$.

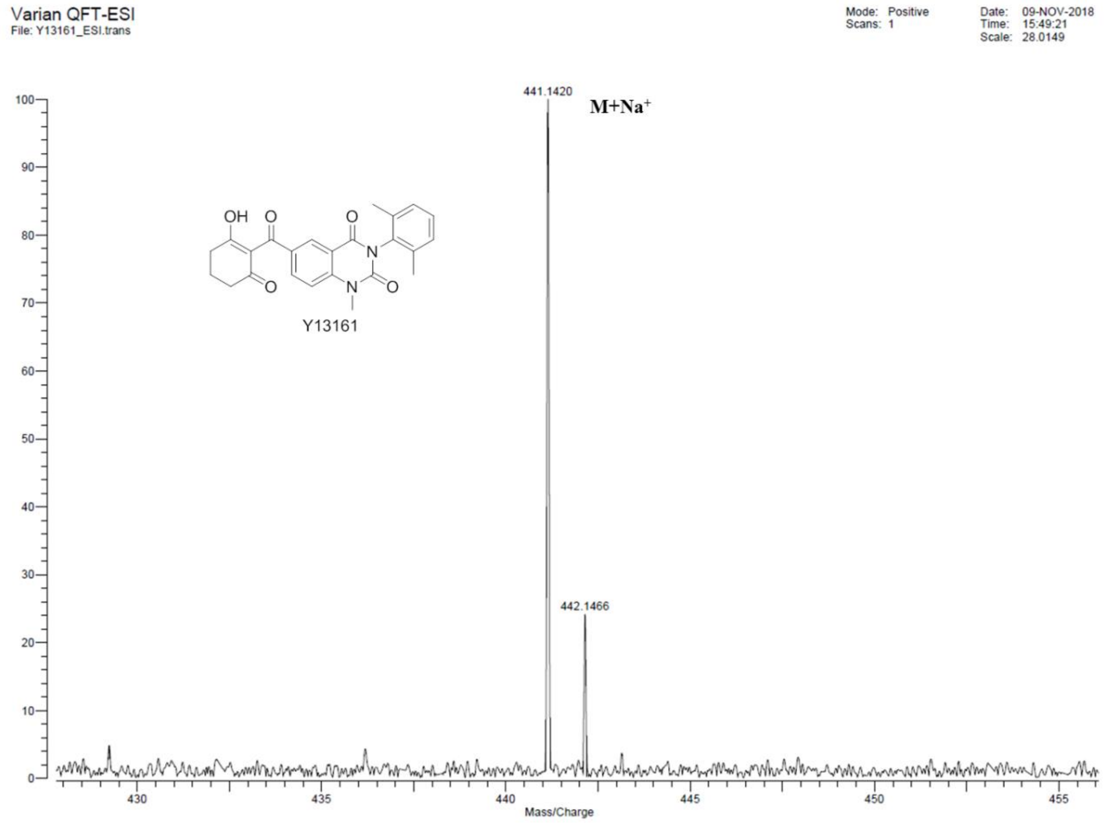

Figure S10 HRMS spectrum of **Y13161**.

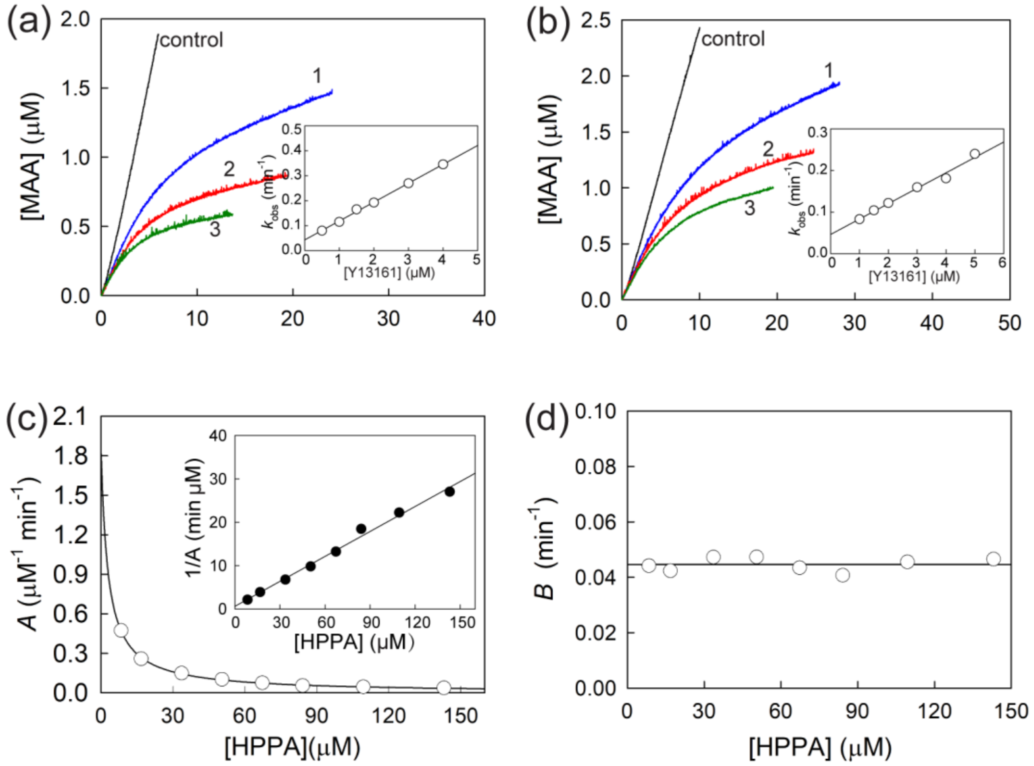

Figure S11 Inhibitory kinetics of *At*HPPD by compound **Y13161**. Each reaction mixture contains 20 mM HEPES (pH 7.0), 2 mM Sodium ascorbate, 100 μM $FeSO_4$, 14 nM *At*HPPD, a certain amount of HPPA ((a) 80 μM; (b) 170 μM), and compound **Y13161** (1, 2.0 μM; 2, 3.0 μM; and 3, 4.0 μM). Experimental data are shown as colored dots and theoretical values as black solid lines. Insets: Plots of k_{obs} against concentration of compound **Y13161**. (c**)** Plot of the apparent rate constant *A* against concentration of HPPA. Inset: Plot of 1/*A* against concentration of HPPA. (d**)** Plot of the apparent rate constant *B* against concentration of HPPA.

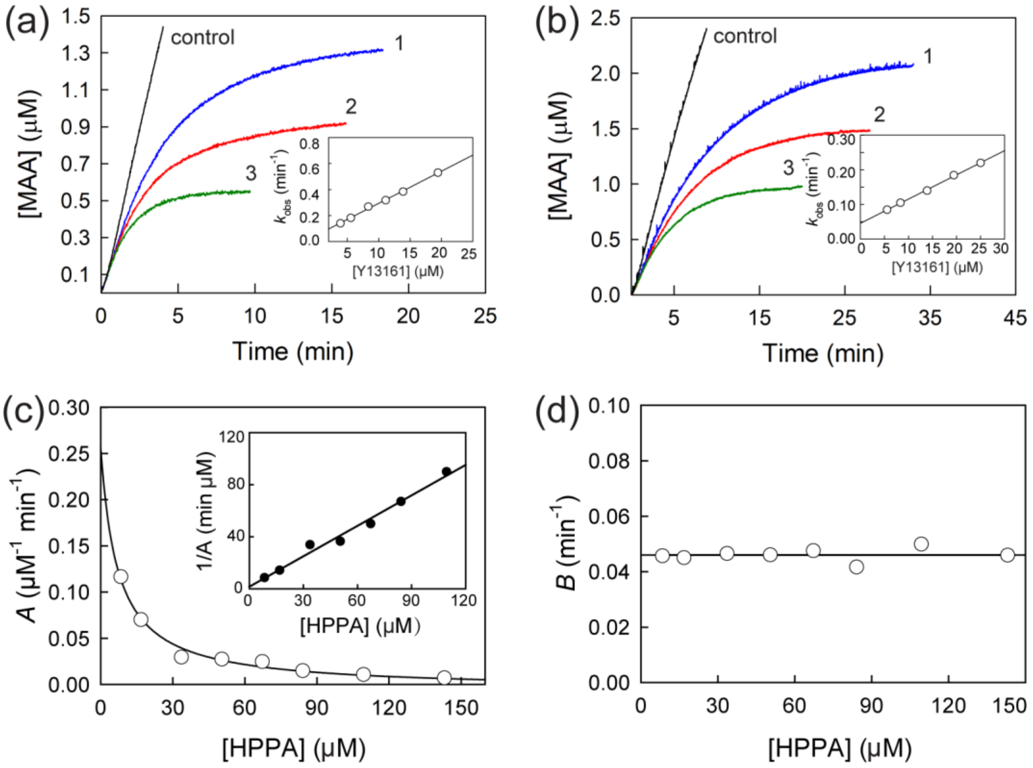

Figure S12 Inhibitory kinetics of *h*HPPD by compound **Y13161**. Each reaction mixture contains 20 mM HEPES (pH 7.0), 2 mM Sodium ascorbate, 100 μM $FeSO_4$, 12 nM *h*HPPD, a certain amount of HPPA ((a) 80 μM; (b) 170 μM), and compound **Y13161** (1, 8.33 μM; 2, 13.88 μM; and 3, 19.44 μM). Experimental data are shown as colored dots and theoretical values as black solid lines. Insets: Plots of *k*obs against concentration of compound **Y13161**. (c**)** Plot of the apparent rate constant *A* against concentration of HPPA. Inset: Plot of 1/*A* against concentration of HPPA. (d**)** Plot of the apparent rate constant *B* against concentration of HPPA.

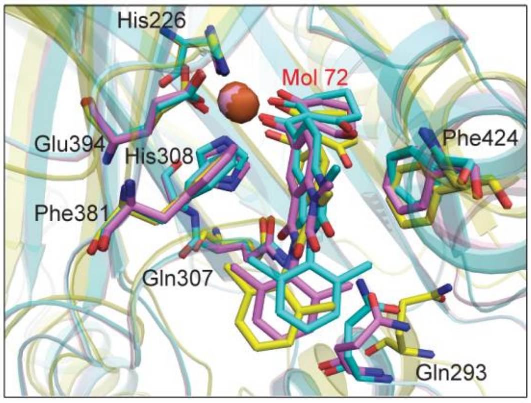

Figure S13 Comparison of docking binding mode (blue) and MD simulated model (yellow)

with co-crystal structure (pink) of *At*HPPD- **Y13161**.

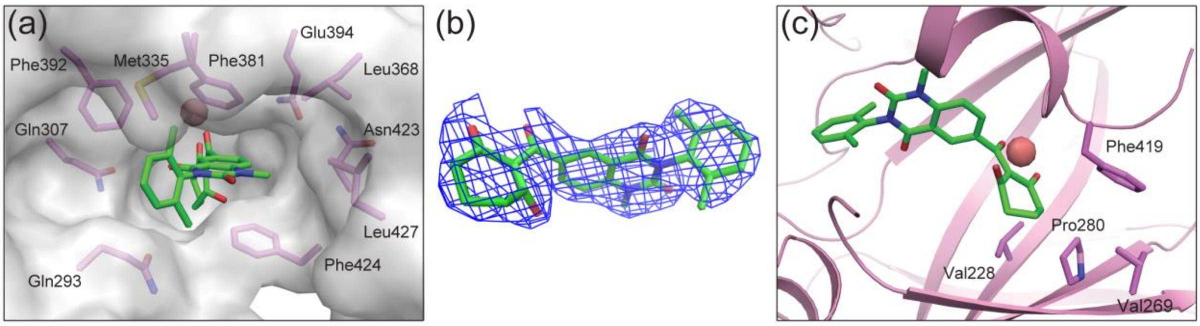

Figure S14 The interactions of **Y13161** with *At*HPPD. (a) Active site pocket of *At*HPPD occupied by Y13161. (b) The 2Fo–Fc map of **Y13161** contoured at 1.0 σ. (c) The hydrophobic interaction of the cyclohexane moiety of **Y13161** with Phe419, Pro280 and Val228.

Supplemental Tables

Table S1 Data collection and refinement statistics for the *At*HPPD-HPPA and *At*HPPD-Y13161 complex.

	*At*HPPD-HPPA	*At*HPPD-Y13161
Crystal parameters		
Space group	P 21	C 1 2 1
a, b, c (Å)	95.60, 95.29, 98.00	77.33, 83.88, 66.31
α, β, γ (º)	90.0, 92.1, 90.0	90.0, 100.1, 90.0
Diffraction data		
Resolution range (Å) [a]	50-2.8 (2.85-2.8) [a]	40-2.4 (2.48-2.40) [a]
Completeness (%) [a]	98.2 (99.8) [a]	95.7 (88.7) [a]
Unique reflections	42692 (4294) [a]	15659 (1474) [a]
*R*merge	0.177 (0.500) [a]	0.092 (0.208) [a]
CC1/2	0.982 (0.828) [a]	0.967 (0.950) [a]
I/σ(I)	6.34 (2.85) [a]	19.7 (5.34) [a]
Subunits per asym. unit	4	1
Refinement statistics		
R_{Work}	0.254 (0.282) [a]	0.193 (0.202) [a]
R_{Free}	0.316 (0.383) [a]	0.245 (0.261) [a]
RMSD Bond length (Å)	0.004	0.004
RMSD Bond angle (º)	0.90	0.61
Clashscore	6.55	3.21
Components of the asymmetry unit (Number of non-hydrogen atoms)		
	two dimers	one monomer
Protein	11102	2828
Substrate or inhibitor	33	32
Waters	212	35
Ramachandran plot (%)		
Favoured	94	97
Outlier	0	0

[a]Numbers in parentheses refer to the highest resolution shell.

Table S2 Top 100 compounds from virtual screening with their rank.

Comp. NO.	Comp. Structure	Comp. NO.	Comp. Structure	Comp. NO.	Comp. Structure
**1**	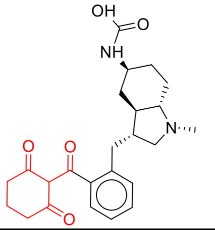	**2**	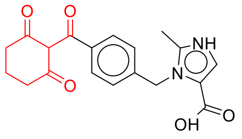	**3**	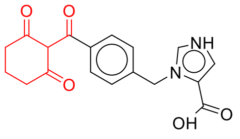
**4**	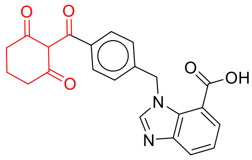	**5**	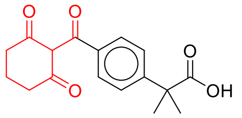	**6**	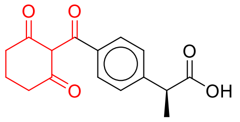
**7**	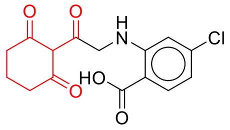	**8**	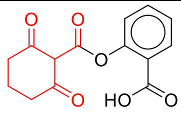	**9**	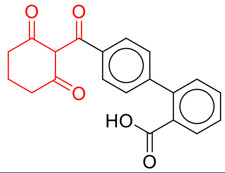
**10**	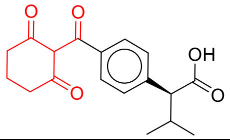	**11**	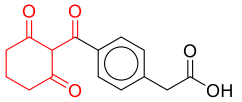	**12**	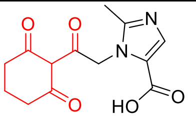
**13**	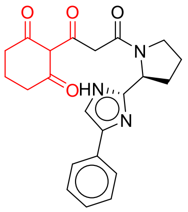	**14**	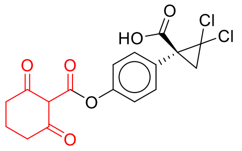	**15**	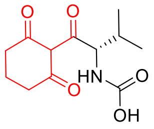
**16**	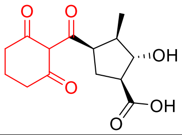	**17**	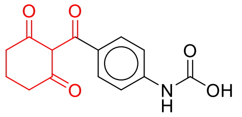	**18**	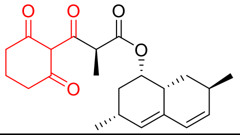
**19**	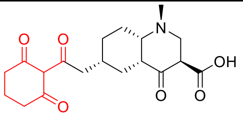	**20**	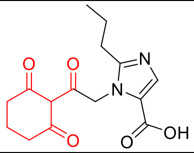	**21**	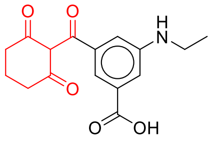
**22**	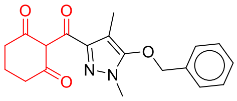	**23**	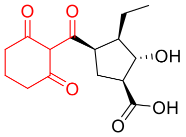	**24**	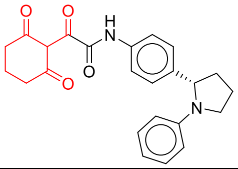
**25**	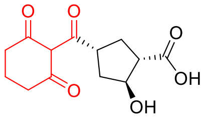	**26**	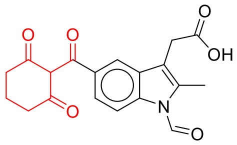	**27**	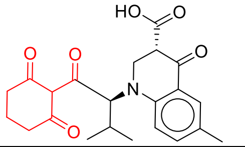

**28**	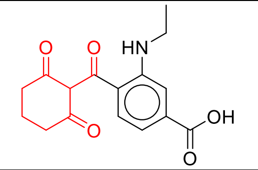	**29**	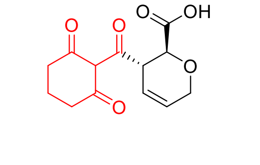	**30**	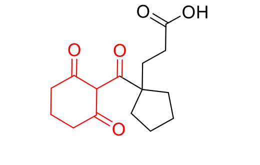
**31**	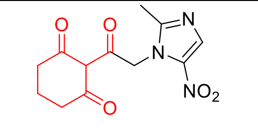	**32**	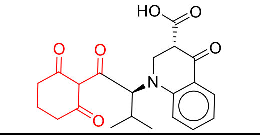	**33**	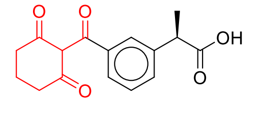
**34**	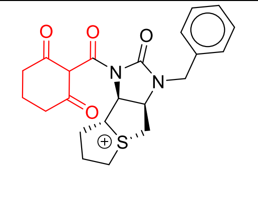	**35**	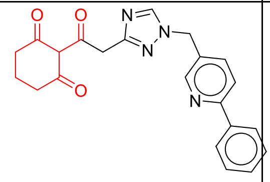	**36**	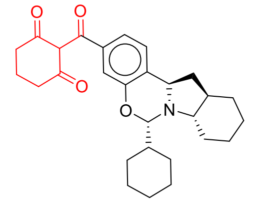
**37**	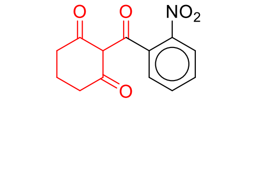	**38**	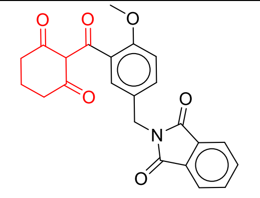	**39**	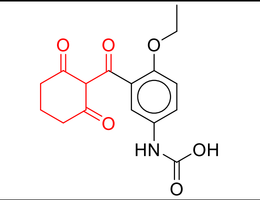
**40**	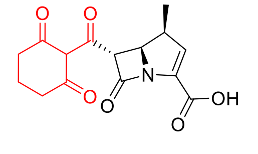	**41**	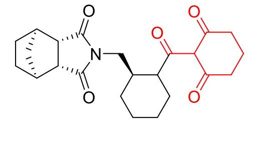	**42**	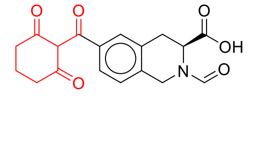
**43**	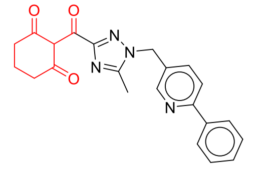	**44**	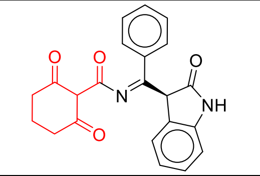	**45**	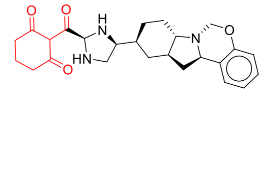
**46**	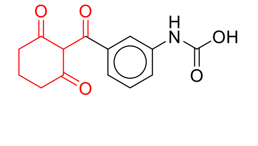	**47**	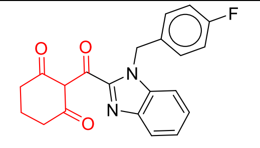	**48**	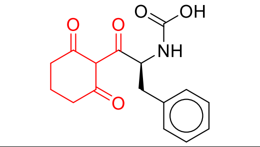
**49**	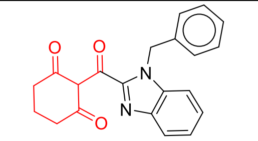	**50**	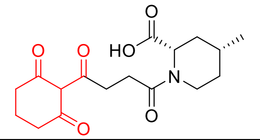	**51**	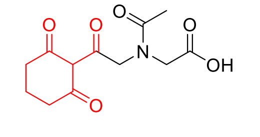
**52**	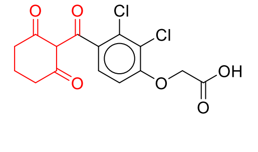	**53**	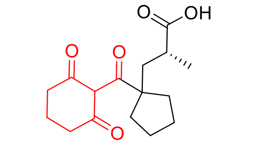	**54**	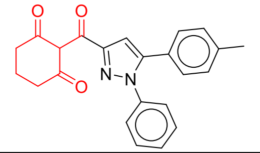
**55**	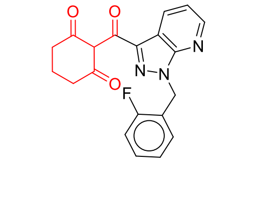	**56**	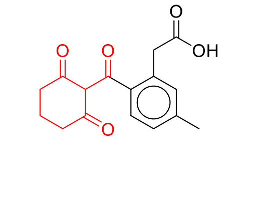	**57**	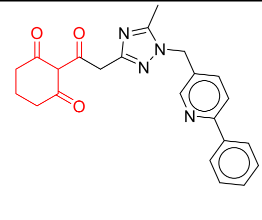

**58**	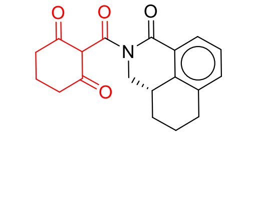	**59**	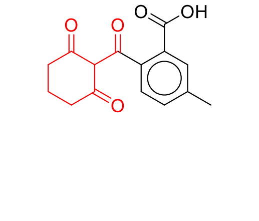	**60**	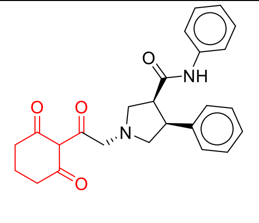
**61**	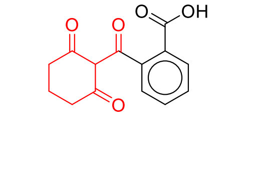	**62**	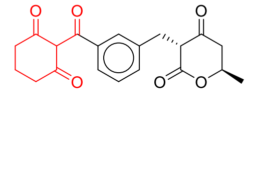	**63**	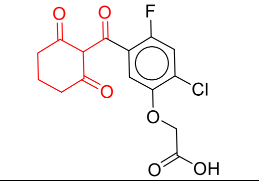
**64**	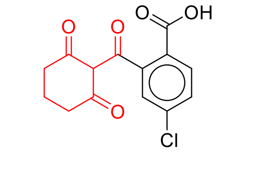	**65**	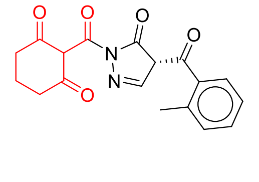	**66**	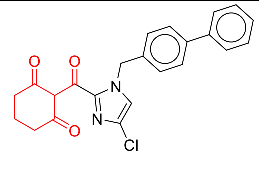
**67**	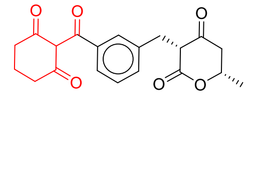	**68**	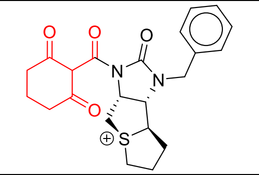	**69**	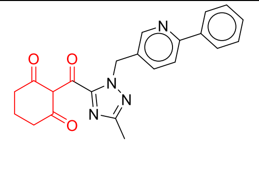
**70**	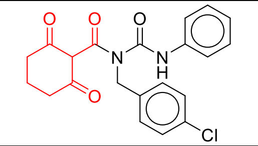	**71**	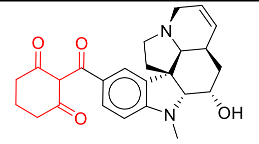	**72**	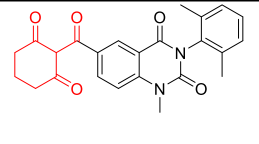
**73**	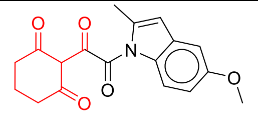	**74**	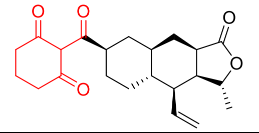	**75**	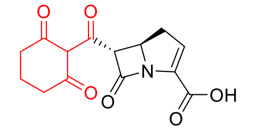
**76**	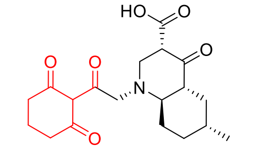	**77**	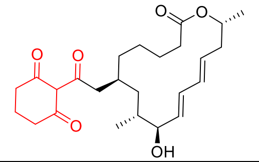	**78**	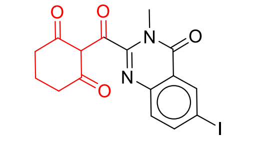
**79**	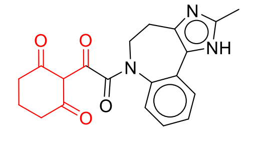	**80**	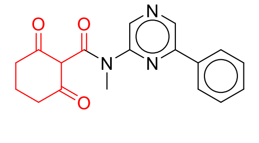	**81**	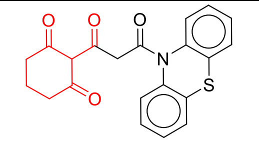
**82**	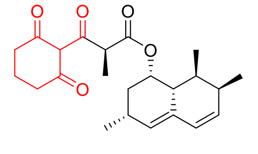	**83**	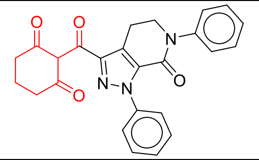	**84**	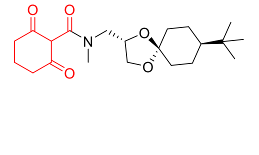
**85**	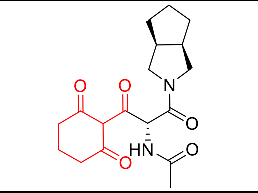	**86**	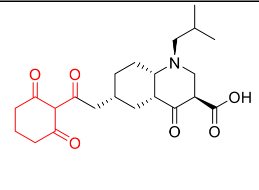	**87**	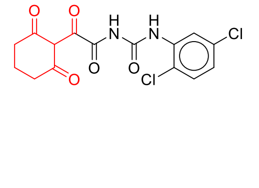
**88**	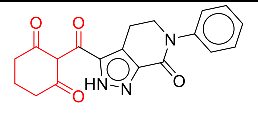	**89**	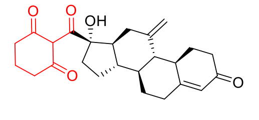	**90**	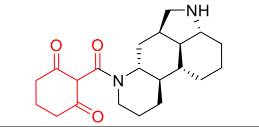

**91**	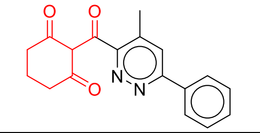	**92**	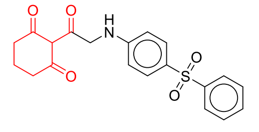	**93**	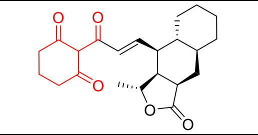
**94**	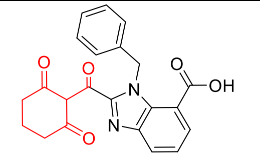	**95**	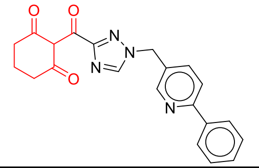	**96**	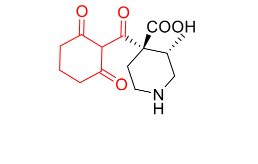
**97**	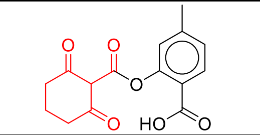	**98**	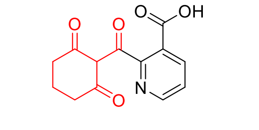	**99**	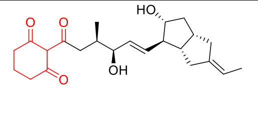
**100**	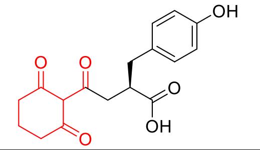				

Table S3 Binding free energy evaluation (kcal/mol) for the top 100 compounds after the structure optimization.

Molecule	H-bond[a]	Electrostatic[b]	vdW[c]	Conformation entropy[d]	Desolvation[e]	Binding free energy
83	-0.01	-1.85	-13.06	1.19	2.49	-11.24
36	-0.02	-1.74	-11.23	0.89	1.43	-10.67
89	0.00	-1.67	-11.04	0.89	1.39	-10.43
24	-0.07	-1.51	-12.35	1.49	2.14	-10.30
66	-0.03	-1.54	-12.59	1.49	2.40	-10.28
43	-0.16	-1.57	-12.34	1.49	2.34	-10.24
93	-0.04	-1.56	-10.74	0.89	1.46	-9.98
57	-0.01	-1.51	-12.23	1.79	2.17	-9.79
71	0.00	-1.09	-11.81	0.89	2.30	-9.71
72	-0.08	-1.57	-11.16	0.89	2.23	-9.69
58	-0.21	-1.37	-10.24	0.30	1.88	-9.64
78	-0.03	-1.58	-10.83	0.60	2.20	-9.64
81	0.00	-1.43	-11.02	0.89	1.96	-9.60

79	-0.46	-1.34	-11.32	0.80	2.74	-9.58
85	-0.03	-1.71	-11.23	1.19	2.33	-9.45
91	-0.02	-1.62	-10.41	0.89	1.79	-9.36
69	-0.12	-1.45	-11.56	1.49	2.28	-9.36
38	-0.27	-2.12	-11.07	1.49	2.82	-9.14
88	-0.58	-1.54	-10.35	0.89	2.44	-9.13
44	-0.06	-1.22	-11.18	0.89	2.49	-9.08
55	0.00	-1.88	-10.84	1.19	2.45	-9.08
18	0.00	-1.17	-10.60	1.49	1.21	-9.07
95	-0.05	-1.48	-11.35	1.49	2.34	-9.06
47	0.00	-1.44	-11.38	1.19	2.64	-8.98
49	-0.01	-1.46	-11.00	1.19	2.38	-8.89
19	-0.04	-3.68	-9.42	1.49	2.82	-8.83
1	-0.01	-1.42	-11.79	1.79	2.60	-8.82
54	0.00	-1.49	-10.31	1.19	1.88	-8.73
92	-0.69	-1.79	-10.25	1.79	2.30	-8.64
76	-0.91	-1.44	-10.54	1.49	2.83	-8.56
9	-0.01	-1.99	-10.81	1.49	2.77	-8.54
87	-0.01	-1.44	-10.59	0.89	2.62	-8.52
35	-0.31	-1.65	-10.82	1.79	2.53	-8.45
90	-0.04	-0.76	-9.49	0.30	1.58	-8.42
94	-0.05	-2.09	-11.01	1.79	3.01	-8.34
42	-0.01	-1.82	-11.35	1.49	3.36	-8.34
73	-0.02	-1.40	-9.94	0.89	2.18	-8.29
77	-0.41	-1.35	-9.34	1.19	1.68	-8.23
4	-0.46	-2.50	-9.89	1.79	2.94	-8.12
33	-0.33	-2.68	-9.11	1.49	2.51	-8.12
67	-0.59	-1.31	-9.25	1.19	1.97	-7.99

68	-0.27	-0.98	-9.47	0.89	1.84	-7.98
75	-0.57	-2.24	-9.04	1.19	2.80	-7.86
26	-0.05	-1.74	-10.92	1.79	3.20	-7.72
39	-0.38	-2.10	-10.48	1.79	3.51	-7.66
34	-0.77	-0.46	-9.60	0.89	2.28	-7.65
16	-0.91	-2.87	-8.11	1.49	2.75	-7.64
80	0.00	-1.54	-9.11	0.89	2.14	-7.63
64	-0.10	-2.30	-9.11	1.19	2.74	-7.59
37	-0.04	-2.77	-8.03	0.89	2.35	-7.59
52	-0.02	-1.83	-10.50	1.79	3.05	-7.50
22	-0.01	-1.27	-9.78	1.49	2.08	-7.49
7	-0.69	-2.19	-9.65	1.79	3.30	-7.44
12	-0.86	-2.76	-8.81	1.49	3.50	-7.44
13	-0.06	-1.76	-9.50	1.49	2.47	-7.36
84	0.00	-1.59	-8.89	1.19	1.95	-7.33
10	0.00	-1.80	-9.44	1.79	2.18	-7.27
5	0.00	-1.79	-9.06	1.49	2.15	-7.21
50	-0.01	-2.25	-8.83	1.79	2.15	-7.16
62	-0.02	-1.58	-8.65	1.19	1.96	-7.10
59	0.00	-2.51	-8.47	1.19	2.70	-7.09
27	-0.90	-2.29	-8.92	1.79	3.24	-7.08
21	-0.53	-2.22	-8.92	1.79	2.81	-7.08
74	0.00	-1.61	-7.38	0.89	1.03	-7.07
100	-0.03	-2.04	-9.83	1.79	3.08	-7.03
82	-0.01	-1.35	-8.52	1.49	1.37	-7.01
23	-1.08	-5.54	-5.37	1.79	3.19	-7.01
6	0.00	-1.81	-9.20	1.49	2.52	-6.99
97	-0.24	-1.94	-9.07	1.49	2.79	-6.97

40	-0.01	-1.71	-9.14	1.19	2.73	-6.93
56	-0.58	-2.02	-8.52	1.49	2.72	-6.92
17	-0.15	-1.79	-9.23	1.19	3.07	-6.91
15	-1.12	-2.12	-8.21	1.49	3.08	-6.88
20	-0.01	-2.45	-9.47	2.09	3.01	-6.84
28	-0.01	-1.96	-9.66	1.79	3.02	-6.82
46	-0.02	-2.14	-8.34	1.19	2.54	-6.76
98	-0.18	-2.04	-8.68	1.19	2.99	-6.71
32	-0.02	-2.48	-8.90	1.79	2.90	-6.71
45	-0.01	0.59	-10.96	0.89	2.80	-6.69
11	-0.01	-1.76	-9.10	1.49	2.70	-6.68
29	-0.71	-2.63	-7.63	1.19	3.16	-6.61
48	-0.32	-1.86	-9.24	1.79	3.05	-6.57
30	-0.34	-2.30	-8.21	1.79	2.56	-6.51
63	-0.45	-1.86	-8.87	1.79	2.89	-6.51
70	0.00	-1.97	-7.99	1.19	2.30	-6.47
51	-1.02	-2.08	-7.98	1.79	2.87	-6.41
53	-0.31	-2.20	-7.72	1.79	2.16	-6.27
60	-0.01	-1.20	-9.27	1.79	2.47	-6.21
65	-0.03	-1.55	-7.47	0.89	1.98	-6.18
31	-0.24	-2.11	-8.00	1.19	2.98	-6.18
86	-0.01	-2.87	-8.42	2.09	3.15	-6.06
14	-0.37	-1.54	-9.14	1.79	3.28	-5.98
3	-0.02	-1.44	-9.66	1.79	3.57	-5.76
2	-0.30	-2.85	-8.17	1.79	3.84	-5.68
25	-0.04	-1.83	-7.65	1.49	2.47	-5.56
8	-0.03	-1.93	-6.68	1.49	2.51	-4.63
61	-0.01	-2.39	-5.89	1.19	2.68	-4.42

41	-0.01	-1.28	-5.05	1.19	1.51	-3.62
96	-0.86	-1.15	-5.92	1.19	3.45	-3.29
99	-0.20	-1.98	-5.23	2.39	1.76	-3.26

[a]Hydrogen bonding term, [b]Electrostatic energies term, [c]van der Waals term, [d]conformation entropy contribution, [e]desolvation contribution.

Table S4 Binding free energy (kcal/mol) calculated for top 10 compounds after structure minimization and MD simulation.

Molecule	H-bond[a]	Electrostatic[b]	vdW[c]	Conformation entropy[d]	Desolvation[e]	Binding free energy
72	-0.05	-1.61	-11.75	1.19	2.52	-9.70
93	-0.79	-1.61	-9.29	0.89	1.59	-9.20
36	-0.02	-1.72	-10.59	1.19	1.96	-9.18
66	-0.02	-1.08	-11.45	1.49	2.17	-8.89
83	-0.23	-1.27	-10.47	1.19	2.19	-8.60
71	-0.12	-1.73	-10.12	1.19	2.23	-8.55
43	0.00	-0.96	-10.69	1.49	1.90	-8.27
57	-0.24	-0.94	-10.41	1.79	1.87	-7.94
24	-0.05	-0.77	-9.70	1.49	1.56	-7.47
89	-0.05	-0.56	-8.91	0.89	1.37	-7.26

[a]Hydrogen bonding term, [b]Electrostatic energies term, [c]van der Waals term, [d]conformation entropy contribution, [e]desolvation contribution.

Table S5 Herbicidal activity of **Y13161** and Mesotrione.

Compd.	Dose (g ai/ha)	EC[a]	SF[a]	DS[a]	AR[a]	EP[a]	AJ[a]
**Y13161**	150	100	100	95	100	70	100
	75	92.5	100	85	100	65	100
	37.5	87.5	100	80	100	55	97.5
Mesotrione	150	85	20	95	100	100	100
	75	75	0	60	100	100	100
	37.5	30	0	30	100	100	100

[a]Abbreviations: EC, Echinochloa crus-galli; SF, Setaria faberii; DS, Digitaria sanguinalis; AR, Amaranthus retroflexu; EP, Eclipta prostrata; AJ, Abutilon juncea.

Table S6 Crop selectivity of **Y13161** and Mesotrione (150 g ai/ha).

Compd.	soybean	rape	cotton	maize	rice	wheat	sorghum
**Y13161**	55	85	30	0	20	10	0
Mesotrione	55	100	70	10	50	40	70

Supplemental methods

Method S1 Preparation of compound Y13161.

All chemical reagents were commercially available and treated with standard methods before use. Solvents were dried and redistilled before use. ^{1}H NMR spectra were recorded on a VARIAN Mercury-Plus 600 or 400 spectrometers in $CDCl_3$ or DMSO-d_6 with TMS as the internal reference, ^{13}C NMR spectra were recorded in $CDCl_3$ on a VARIAN Mercury-Plus 400 (101 MHz) spectrometer, and chemical shifts (δ) are given in ppm relative to the centre line of a triplet at 77.0 ppm of $CDCl_3$. The following abbreviations are used to designate multiplicities: s = singlet, d = doublet, t = triplet, m = multiplet, br = broad. High resolution mass spectra (HRMS) were obtained on an Agilent 6224 TOF LC/MS (USA). Melting points were taken on a Buchi B-545 melting point apparatus and are uncorrected.

Scheme 1. Synthetic route of compound **Y13161**.

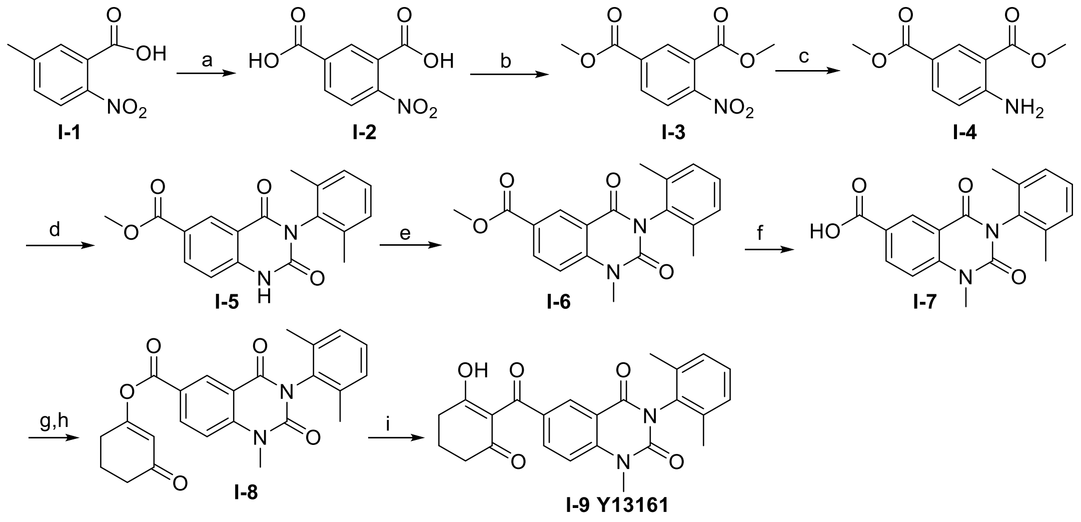

Reagents and conditions: (a) KOH, $KMnO_4$, HCl; (b) CH_3OH, H_2SO_4, reflux; (c) H_2, 10% Pd/C; (d) 2-isocyanato-1,3-dimethylbenzene, Pyridine, 100 ℃; (e) Cs_2CO_3, iodomethane, DMF, rt; (f) Sulfuric acid, acetic acid, H_2O; (g) $SOCl_2$, THF, reflux; (h) 1,3-cyclohexanediones, Et_3N, $CHCl_3$, 0 ℃; (i) Acetone cyanohydrin, Et_3N, CH_2Cl_2, rt.

Synthesis of 4-nitroisophthalic acid I-2. To a three neck 2500 mL round-bottom flask equipped with a mechanical stirrer and a reflux condenser were added 5-methyl-2-nitrobenzoic acid (100 g, 553 mmol) and water (1000 mL). KOH (31 g, 553 mmol)

was added with stirring; after the reaction mixture became clear, the solution was heated to 90 ℃ and KMnO4 (262.2 g, 1659 mmol) was added portion-wise over about 1 h. The suspension was then heated at this temperature for another 3 h, the reaction medium was filtered, and the residue was washed with hot water (100 mL) for three times. The filtrate was cooled to room temperature and acidified with concentrated HCl to pH = 1~2. The resulting white solid was collected by filtration and washed with water (100 mL) for three times, then dried to give **I-2** as a white solid (105 g, yield 90 %). mp, 244-246 ℃; ^{1}H NMR (600 MHz, DMSO-*d*6) δ 13.99 (brs, 2H), 8.34 (d, J = 1.2 Hz, 1H), 8.27 (dd, J = 8.4, 1.8 Hz, 1H), 8.08 (d, J = 8.4 Hz, 1H).

Preparation of dimethyl 4-nitroisophthalate I-3. To a three neck 1000 mL round-bottom flask equipped with a mechanical stirrer and a reflux condenser were added 4-nitroisophthalic acid **I-2** (100 g, 474 mmol) and methanol (500 mL). Concentrated H_2SO_4 (30 mL) was added drop-wise to the suspension over 30 min. The resulting solution was heated to reflux overnight, and the methanol was then removed under reduced pressure. After cooling to room temperature, the resulting white solid was dissolved in 900 mL EtOAc, the organic phase was washed with H_2O (200 mL) for three times, then with saturated aqueous $NaHCO_3$ (200 mL) for three times, and finally with saturated brine (200 mL) for three washes. The organic layer was dried by anhydrous Na_2SO_4 and concentrated by rotary evaporation to give **I-3** as a white solid (107.6 g, yield 95%). mp, 84-86 ℃; ^{1}H NMR (600 MHz, $CDCl_3$) δ 8.44 (s, 1H), 8.29 (d, J = 8.4 Hz, 1H), 7.93 (d, J = 8.4 Hz, 1H), 3.99 (s, 3H), 3.95 (s, 3H).

Preparation of dimethyl 4-aminoisophthalate I-4. To a solution containing dimethyl 4-nitroisophthalate 3 (100 g, 419 mmol) in 800 mL EtOAc was added 10 g of 10% Pd/C. The mixture was hydrogenated at normal pressure for 20 h. After the reaction was completed according to TLC detection, the reaction medium was filtered through a bed of Celite, and the residue was washed with EtOAc (50 mL) for three times. After removal of the solvent under reduced pressure, **I-4** was obtained as a white solid (84.8 g, yield 97%). mp, 127-129 ℃; ^{1}H NMR (400 MHz, CDCl3) δ 8.59 (d, J = 1.6 Hz, 1H), 7.91 (dd, J = 8.4 Hz, 2.0 Hz, 1H), 6.66 (d, J = 8.8 Hz, 1H), 6.28 (brs, 2H), 3.90 (s, 3H), 3.88 (s, 3H).

Synthesis of methyl 3-(2,6-dimethylphenyl)-2,4-dioxo-1,2,3,4-tetrahydroquinazoline-6-carboxylate I-5. Dimethyl 4-aminoisophthalate **I-4** (20 mmol) and pyridine (30 mL) were added to a two neck 100 mL round-bottom flask and 2-isocyanato-1,3-dimethylbenzene (25 mmol) was added with stirring. The resulting solution was heated to 100 ℃ under N2 atmosphere for about 6 h. After completion of the reaction according to TLC detection, the reaction solution was cooled to room temperature and poured into water (100 mL). The mixture was stirred vigorously for 30 min and during this process a solid was formed. The resulting solid was collected by filtration and washed with ether (50 mL), then dried under vacuum to afforded **I-5** in yield of 84%, mp 257-259 ℃. ^{1}H NMR (400 MHz, DMSO-*d*6) δ 12.10 (s, 1H), 8.52 (d, *J* = 2.0 Hz, 1H), 8.26 (dd, *J* = 8.4, 2.0 Hz, 1H), 7.37 (d, *J* = 8.4 Hz, 1H), 7.29–7.24 (m, 1H), 7.20 (d, *J* = 7.2 Hz, 2H), 3.88 (s, 3H), 2.03 (s, 6H).

Preparation of methyl 3-(2,6-dimethylphenyl)-1-methyl-2,4-dioxo-1,2,3,4-tetrahydroquinazoline-6-carboxylate I-6. Compounds **I-5** (15 mmol) and DMF 75 mL were added into a single neck round bottom flask, and Cs_2CO_3 (18 mmol) was added to the solution with stirring. After stirring at room temperature for 30 min, methyl iodide (30 mmol) was added to the mixture and the reaction mixture was then stirred for another 6-24 h. After completion of the reaction according to the TLC detection, the reaction mixture was poured into water (300 mL), and stirred vigorously for 30 min. The resulted solid was collected by filtration and washed with water (50 mL), then dried under vacuum to afforded **I-6** in yield of 81%, mp 234-236 ℃. ^{1}H NMR (600 MHz, DMSO-*d*6) δ 8.61 (s, 1H), 8.35 (d, *J* = 9.0 Hz, 1H), 7.69 (d, *J* = 8.4 Hz, 1H), 7.29–7.23 (m, 1H), 7.20 (d, *J* = 7.2 Hz, 2H), 3.90 (s, 3H), 3.61 (s, 3H), 2.02 (s, 6H).

Synthesis of 3-(2,6-dimethylphenyl)-1-methyl-2,4-dioxo-1,2,3,4-tetrahydroquinazoline-6-carboxylic acid I-7. I-6 (10 mmol), HOAc (100 mL), and water (50 mL) was added into a single neck 500 mL round bottom flask, and H_2SO_4 (50 mL) was added into the mixture over 20 min. The suspension was then heated to 100 ℃ for 12 h, until the reaction was completed according to TLC detection. The reaction medium was cooled to room temperature, poured into ice-cold water (500 mL) and stirred for 30 min. The resulting

solid solid was collected by filtration and washed with water (50 mL) and dried in vacuo to afford **I-7** in yield of 95%, mp 269-271 ℃. ^{1}H NMR (600 MHz, DMSO-*d6*) δ 13.29 (brs, 1H), 8.60 (d, *J* = 1.2 Hz, 1H), 8.33 (dd, *J* = 8.4, 1.2 Hz, 1H), 7.67 (d, *J* = 8.4 Hz, 1H), 7.26 (t, *J* = 7.2 Hz, 1H), 7.20 (d, *J* = 7.2 Hz, 2H), 3.61 (s, 3H), 2.02 (s, 6H).

Synthesis of 3-oxocyclohex-1-en-1-yl 3-(2,6-dimethylphenyl)-1-methyl-2,4-dioxo-1,2,3,4-tetrahydroquinazoline-6-carboxylate I-8. I-7 (2 mmol) and THF (40 mL) were added into a single neck flask, two drops of DMF was added to the mixture, and $SOCl_2$ (3 mmol) was added to the solution over 10 min with stirring. The suspension was then heated to reflux for 3 h. The solvent of the reaction was removed under reduced pressure to afford the acid chloride; the acid chloride thus obtained was then dissolved in $CHCl_3$ (20 mL). The solution was added drop-wise to a solution of cyclohexane-1,3-dione (2 mmol) and Et3N (4 mmol) in $CHCl_3$ (20 mL) at 0 ℃. The mixture was then stirred at room temperature for 1 h, until the reaction was completed according to TLC detection. Water (50 mL) was added to the solution, and the mixture was stirred vigorously for 30 min. The organic layer was washed by aqueous HCl solution (50 mL, 1 mol/L), saturated aqueous $NaHCO_3$ (50 mL) and brine (50 mL) in this order, dried by anhydrous Na_2SO_4, and concentrated by rotary evaporation. The residue was purified via flash chromatography to give intermediate **I-8** in yield of 75%, mp 167-169 ℃. ^{1}H NMR (600 MHz, $CDCl_3$) δ 8.97 (s, 1H), 8.42 (d, *J* = 9.0 Hz, 1H), 7.41 (d, *J* = 9.0 Hz, 1H), 7.29 (d, *J* = 7.2 Hz, 1H), 7.21 (d, *J* = 7.2 Hz, 2H), 6.09 (s, 1H), 3.73 (s, 3H), 2.57 (s, 2H), 2.34 (s, 2H), 2.12 (s, 6H), 1.17 (s, 6H).

Preparation of 3-(2,6-dimethylphenyl)-6-(2-hydroxy-6-oxocyclohex-1-ene-1-carbonyl)-1-methylquinazoline-2,4(1H,3H)-dione (Y13161). Compound **I-8** (1 mmol) was dissolved in anhydrous CH_2Cl_2 (30 mL) with stirring and Et_3N (2 mmol) and acetone cyanohydrin (0.1 mmol) were added into the solution; the mixture was then stirred at room temperature under N_2 protection for 12 h. The progress of the reaction to completion was followed by TLC detection. The organic layer was washed with aqueous HCl solution (30 mL, 1 mol/L) for three times, and brine (30 mL) for two times, dried by anhydrous Na_2SO_4 and then concentrated by rotary evaporation. The residue was purified via flash chromatography to give compound **I-9** in yield of 90%, mp, 187-189 ℃; ^{1}H NMR (600 MHz, $CDCl_3$) δ 16.83

(s, 1H), 8.45 (d, J = 1.8 Hz, 1H), 7.90 (dd, J = 9.0, 1.8 Hz, 1H), 7.29 (d, J = 9.0 Hz, 1H), 7.24 (d, J = 7.2 Hz, 1H), 7.18 (d, J = 7.2 Hz, 2H), 3.69 (s, 3H), 2.78 (t, J = 6.0 Hz, 2H), 2.52 (t, J = 6.6 Hz, 2H), 2.14–2.06 (m, 8H). ^{13}C NMR (101 MHz, $CDCl_3$) δ 196.52, 196.38, 194.30, 160.24, 149.89, 143.26, 135.50, 135.27, 133.59, 132.94, 130.20, 128.83, 128.48, 114.87, 112.99, 37.91, 32.18, 31.05, 18.91, 17.71. ^{13}C NMR (101 MHz, $CDCl_3$) δ 196.35, 196.26, 194.18, 160.12, 149.72, 143.13, 135.42, 135.15, 133.53, 132.79, 129.99, 128.65, 128.32, 114.70, 112.94, 112.87, 37.76, 32.01, 30.92, 18.77, 17.57. HRMS (ESI): calcd for $C_{24}H_{22}N_2O_5$ $[M^{+}Na]^{+}$ 441.1426, found: 441.1420.

Method S2 Inhibitory Kinetics of HPPD.

Scheme S2. The reaction mechanism for the competitive slow-binding inhibitors.

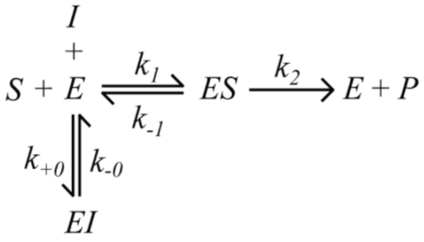

Where S, E, I and P represent the substrate, enzyme, inhibitor and product, respectively. According to the substrate reaction kinetic theory, the accumulation of product with time can be expressed by equation (1):

$$[P] = v_s t + \frac{v_0 - v_s}{k_{obs}}\left(1 - e^{-k_{obs} t}\right) \quad (1)$$

where v_0 and v_s are the initial and steady-state velocities of the reaction in the presence of inhibitor. k_{obs} is the observed first order rate constant, which can be generated against inhibitor concentration.

$$k_{obs} = A[I]_0 + B \quad (2)$$

Experimentally, the association and dissociation rate constants k_{+0} and k_{-0} can be ascertained by studying the effect of [S] on the apparent rate constants A and B.

$$A = \frac{k_{+0}}{1+\frac{[S]}{K_m}} \tag{3}$$

$$B = k_{-0} \tag{4}$$

where K_m is Michaelis-Menten constants.

Method S3 Computational Simulation.

Structure-based virtual screening. Among the commercialized HPPD herbicides, six of them belongs to the triketone derivatives and they are the most deeply studied, owning to their structure diversity. So we constructed a triketone-linked molecules library by using an in house fragment library based on the fragment-based drug design (FBDD) strategy.[1] The library consists of three-dimensional structures of 9,402 medicine fragments and 5,833 pesticide fragments. The three-dimensional structure of motif 2-benzoylcyclohexane-1,3-dione were constructed with SYBYL 7.0[2] as the core and then linked it to all the fragments by using a modified version of AutoGrow program.[3] Finally, we got the molecule library, that contains 15,235 triketone derivatives for virtual screening. The structure of *At*HPPD was taken from the Protein Data Bank (PDB ID 1SQD) and was prepared with Discovery Studio 2.5 software.[4] A consensus docking strategy was performed to get binding pose for every library molecule. AutoDock 4.0,[5] Vina 1.1.2,[6] Plants 1.2,[7] LeDock[8] were used to search binding conformations for molecules at the iron(II) active center of *At*HPPD. For every docking tools we got 20 conformations and then all of them were clustered by 0.8 Å of RMSD criteria. During the docking process, Gln293 representative conformations were selected from every cluster and the semiempirical score function in AutoDock4.0 was used to evaluate binding free energy for the ligand-*At*HPPD system. The best scored conformation was taken into account as the final docking pose. Finally, we got the binding energy ranking list for 14,751 molecules after excluding some invalid data (work flow can been see in main text Fig. 4).

Structure optimization and MD simulation. The top 100 structures (Table S2) of 14,751 result were selected out for further study. Three-step energy minimization were carried out to every selected ligand-*At*HPPD complex by using Sander of Amber16 program,[9] first to minimize all the hydrogens and other atoms were fixed. Secondly, only backbone atoms of HPPD were fixed, and others were allowed to move. Thirdly, all atoms were free to move. For all the three steps, we used steepest descent method for 2000 steps and conjugated gradient method for 2000 steps. The binding free energy between the top 100 molecules and *At*HPPD was recalculated based on the optimized structures. The results were shown in Table S3 ranked by the value of binding free energy. To further confirm the binding stability, molecular dynamics (MD) simulation was performed for the 10 best bound candidates (number 83, 36, 89, 24, 66, 43, 93, 57, 71, 72) of the 100 molecules with the *At*HPPD. For MD simulationm, the quantum mechanics (QM) calculations were first performed for the 10 candidates at the HF/6-31+G* basis function to obtain the electrostatic potential by using the restrained electrostatic potential (RESP) method.[10] Then, Antechamber module in Amber16 program was employed to generate RESP charges for the molecules. The optimized structures in previous step were used as initial ligand-*At*HPPD complex structure for MD simulation and the topology and coordinate files were constructed with Leap module in Amber16 program under ff14SB force field.[11] Each complex was solvated in the TIP3P waters[12] and neutralized by the counterions. 50 ps's simulation was first added to the solvent molecules and ions for getting an equilibrated solvent environment. Then the system temperature was heated from 0 K to 298 K during 100 ps. At last, 6 ns's simulation was maintained at 298 K with a constant pressure. During the MD simulation, we used a distance constraint setting to make the bidentate association between active site Fe(II) and oxygens on ligand triketone motif keep a reasonable distance. The periodic boundary condition and SHAKE algorithm[13] were also applied for the MD simulation. The plot of root-mean-square deviation (RMSD) of the protein backbone and ligand atoms across the whole MD process was examined for convergence (Fig. S13). We can find that all the five candidates can reach equilibrium states according to the RMSD values of the MD trajectory. For a more precise examination of binding free energy, 100 snapshots for every of the 10 compounds were extracted from the last 1ns MD trajectory

with a time interval of 10 ps by using Cpptraj module[14] in Amber16 program. The average binding free energy were calculated by using the AutoDock semiempirical score function. The binding free energy (Table S3) for the 10 candidates range from -6.80 kcal/mol to -9.70 kcal/mol. Molecule 72 shows the best binding affinity with the value -9.70 kcal/mol. Compared the energy terms between molecule 72 and others, the mainly difference comes from the van der Waals (vdW) energy term, that means molecule 72 has stronger vdW interaction with HPPD than the others. The co-crystal structure of molecule 72 bind with *At*HPPD were resolved by us, and it is used to compare with the docking binding mode and MD convergent conformation (Fig. S14). We can see that the docking conformation (blue) and MD convergent conformation (yellow) of molecule 72 keep very similar binding pose with the crystal conformation (green).

Supplemental Reference

[1] Hao GF, Jiang W, Ye YN, Wu FX, Zhu XL, Guo FB, Yang GF (2016) ACFIS: a web server for fragment-based drug discovery. *Nucleic Acids Res* **44** (W1): W550-W556. doi: 10.1093/nar/gkw393.

[2] Molecular Modeling System SYBYL, TROPS Associates, Inc., St. Louis, MO 63117.

[3] Durrant JD, Amaro RE, McCammon JA (2009) AutoGrow: a novel algorithm for protein inhibitor design. *Chem Biol Drug Des* **73** (2): 168-178. doi: 10.1111/j.1747-0285.2008.00761.x.

[4] Dassault Systèmes BIOVIA, Discovery Studio Modeling Environment, Release 2017, San Diego: Dassault Systèmes, 2016.

[5] Huey R, Morris GM, Olson AJ, Goodsell DS (2007) A semiempirical free energy force field with charge-based desolvation. *J Comput Chem* **28** (6): 1145-1152. doi: 10.1002/jcc.20634.

[6] Trott O, Olson AJ (2010) AutoDock Vina: improving the speed and accuracy of docking with a new scoring function, efficient optimization, and multithreading. *J Comput Chem* **31** (2): 455-461. doi: 10.1002/jcc.21334.

[7] Korb O, Stutzle T, Exner TE (2009) Empirical scoring functions for advanced protein-ligand docking with PLANTS. *J Chem Inf Model* **49** (1): 84-96. doi: 10.1021/ci800298z.

[8] Wang Z, Sun H, Yao X, Li D, Xu L, Li Y, Tian S, Hou T (2016) Comprehensive evaluation of ten docking programs on a diverse set of protein-ligand complexes: the prediction accuracy of sampling power and scoring power. *Phys Chem Chem Phys* **18** (18): 12964-12975. doi: 10.1039/c6cp01555g.

[9] Case, D.A., Betz, R.M., Cerutti, D.S. & Kollman, P.A. (2016) AMBER 2016:University of California, San Francisco.

[10] Singh, U.C. & Kollman, P.A. (1984) An approach to computing electrostatic charges for molecules. *J Comput Chem* **5** (2): 129-145.

[11] Maier JA, Martinez C, Kasavajhala K, Wickstrom L, Hauser KE, Simmerling C (2015) ff14SB: Improving the Accuracy of Protein Side Chain and Backbone Parameters from ff99SB. *J Chem Theory Comput* **11** (8): 3696-3713. doi: 10.1021/acs.jctc.5b00255.

[12] Jorgensen, W.L., Chandrasekhar, J., Madura, J.D., Impey, R.W. & Klein, M.L. (1983) Comparison of simple potential functions for simulating liquid water. *J Chem Phys* **79**: 926-935.

[13] Ryckaert, J.P., Ciccotti, G. & Berendsen, H.J.C. (1977) Numerical integration of the Cartesian equations of motion of a system with constraints: molecular dynamics of n-alkanes. *J Comput Phys* **23** (3): 327-341.

[14] Roe DR, Cheatham TE, 3rd (2013) PTRAJ and CPPTRAJ: Software for Processing and Analysis of Molecular Dynamics Trajectory Data. *J Chem Theory Comput* **9** (7): 3084-3095. doi: 10.1021/ct400341p.
